# Natural Product-Based Potential Therapeutic Interventions of Pulmonary Fibrosis

**DOI:** 10.3390/molecules27051481

**Published:** 2022-02-22

**Authors:** Mahbub Hasan, Nidhan Chandra Paul, Shamrat Kumar Paul, Abu Saim Mohammad Saikat, Hafeza Akter, Manoj Mandal, Sang-Suk Lee

**Affiliations:** 1 Department of Biochemistry and Molecular Biology, Life Science Faculty, Bangabandhu Sheikh Mujibur Rahman Science and Technology University, Gopalganj, Dhaka 8100, Bangladesh; ncpaul73@gmail.com (N.C.P.); shamratpaul.bmb@gmail.com (S.K.P.); asmsaikat.bmb@gmail.com (A.S.M.S.); manojju2008@gmail.com (M.M.); 2 Department of Oriental Biomedical Engineering, College of Health Sciences, Sangji University, Wonju 26339, Korea; 3 Pharmacology and Toxicology Research Division, Health Medical Science Research Foundation, Dhaka 1207, Bangladesh; shemuli.bio86@gmail.com

**Keywords:** pulmonary fibrosis, natural products, therapeutic targets, plant active compounds, plant extracts, herbal medicine

## Abstract

Pulmonary fibrosis (PF) is a disease-refractive lung condition with an increased rate of mortality. The potential factors causing PF include viral infections, radiation exposure, and toxic airborne chemicals. Idiopathic PF (IPF) is related to pneumonia affecting the elderly and is characterized by recurring scar formation in the lungs. An impaired wound healing process, defined by the dysregulated aggregation of extracellular matrix components, triggers fibrotic scar formation in the lungs. The potential pathogenesis includes oxidative stress, altered cell signaling, inflammation, etc. Nintedanib and pirfenidone have been approved with a conditional endorsement for the management of IPF. In addition, natural product-based treatment strategies have shown promising results in treating PF. In this study, we reviewed the recently published literature and discussed the potential uses of natural products, classified into three types—isolated active compounds, crude extracts of plants, and traditional medicine, consisting of mixtures of different plant products—in treating PF. These natural products are promising in the treatment of PF via inhibiting inflammation, oxidative stress, and endothelial mesenchymal transition, as well as affecting TGF-β-mediated cell signaling, etc. Based on the current review, we have revealed the signaling mechanisms of PF pathogenesis and the potential opportunities offered by natural product-based medicine in treating PF.

## 1. Introduction

Pulmonary fibrosis (PF) is defined as a diverse set of lung illnesses manifested by the gradual and permanent deterioration of the lung architecture, induced by scar formation, which eventually results in organ dysfunction, interruption of gas exchange, and mortality from respiratory failure [1,2]. IPF is a chronic, recurrent form of fibrosing interstitial pneumonia that primarily affects elderly persons and is restricted to the lungs. Considering the growing attention to the pathophysiology of IPF, the disease continues to have a poor prognosis [3,4]. Moreover, IPF is a very aggressive type of pulmonary fibrosis with an unclear etiology and a 2–6-year survival rate following diagnosis [3,5]. After viral infection or exposure to chemotherapeutic medications, radiotherapy, or environmental toxins, PF can also progress [6,7]. Additionally, PF develops in multiple bone marrow transplant patients who have continuous grafts against host disease and in a group of people who have long-term inflammatory disorders such as rheumatoid arthritis or scleroderma [8]. Reportedly, lung transplantation is an efficient treatment for developing lung fibrosis.

Even though PF can progress with the deficiency of a specific initiating factor and can reach a clinically apparent acute inflammatory stage, it is more frequently linked with significant lung damage triggered by respiratory infections, persistent granulomatous illnesses, drug side effects, or connective tissue abnormalities [9,10]. In addition, PF is a vital component of the pathogenesis of MERS and SARS, as demonstrated by clinical, autopsy, and radiographic evidence [11,12,13]. Given the lack of a documented, effective, and specific treatment for pulmonary fibrosis, risk mitigation efforts are implemented to reduce the intensity of the infection and to shield the lung from additional accidental damage.

The promising anti-PF role of natural compounds has garnered growing interest over the last two decades, although it remains poorly understood. The potential benefits of natural products include their numerous biological properties and robust safety standards, which are essential elements in the prevention and treatment of pulmonary fibrosis [14].

## 2. Detailed Pathogenesis of Pulmonary Fibrosis

PF refers to a category of lung diseases distinguished by the gradual and permanent deterioration of the lung infrastructure triggered by scar development, which gradually contributes to organ dysfunction [15,16], the disturbance of gas exchange [17,18], and mortality due to respiratory impairment [19]. IPF is a persistent, systemic lung condition defined by recurrent lung scarring and a histological image similar to the usual image of interstitial pneumonia. PF is connected to a worsening cough and dyspnea and reduced life expectancy [7,20]. PF can also occur following viral infections and exposure to radiation, airborne toxic chemicals, and chemotherapeutic drugs [21,22,23,24]. Lung transplantation is presently the most effective treatment for progressive lung fibrosis. The British Thoracic Society made a weak recommendation for the use of prednisolone, N-acetylcysteine, and azathioprine for treating PF [25]. Nintedanib and pirfenidone were authorized, with a conditional endorsement, for the management of IPF in 2014 [26]. A recent study demonstrated that nintedanib and pirfenidone had similar and favorable safety profiles, whereas N-acetylcysteine showed an elevated risk of adverse effects [27].

**Figure 1:**
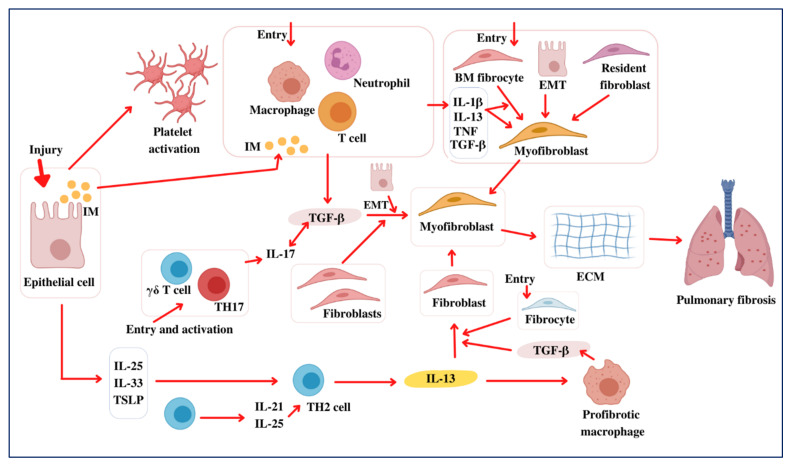
Pathogenesis of pulmonary fibrosis. Following lung injury, epithelial cells unleash inflammatory mediators (IMs) that activate an antifibrinolytic coagulation mechanism, resulting in platelet stimulation and blood clot initiation. The activated leukocytes secrete profibrotic cytokines, including TNF, IL-1β, IL-13, and TGF-β. Additionally, this enables neutrophils and macrophages to expel dead cells and any aggressive organisms. Subsequently, bone marrow (BM) fibrocytes and resident fibroblasts propagate and develop into myofibroblasts and secrete ECM elements. Furthermore, fibroblasts and myofibroblasts may be produced from epithelial cells that have undergone EMT. The myofibroblasts further facilitate wound healing during the ultimate redecorating and resolution process, resulting in wound contraction and blood vessel regeneration. Fibrosis often occurs if some phase of the tissue regeneration process is poorly developed or if the lung-damaging stimuli continue. TGF-β also acts on epithelial cells, causing EMT and the development of the myofibroblasts that produce ECM. TGF-β1 aggravates the inflammatory activity further by inducing Th17 cell differentiation, leading to PF. Likewise, the epithelial cells produce IL-33, IL-25, and TSLP in response to damage, promoting the production of profibrotic Th2 responses. T cells also have IL-25 and IL-21, which facilitate Th2 differentiation. The Th2 cells produce IL-13, enabling a profibrotic macrophage subpopulation (PMS) that releases TGF-β1 and other mediators. Additionally, unaided IL-13 can actively trigger the fibroblasts of TGF-β1. Consequently, Th2 cytokines induce unique chemokines that facilitate the activity of collagen-secreting fibrocytes (CSFs) from the bone marrow (BM), amplifying the fibrotic responses. As a result, myofibroblasts form to unlock ECM elements and, therefore, lead to PF development.

Repair of injured tissues is a necessary biological feature that enables orderly regeneration for defective or diseased cells [28]. Nevertheless, there are cases in which this mechanism appears to be poorly developed. In that case, it may result in the formation of a perpetual fibrotic “scar” at the source of tissue damage, which is defined by an irregular aggregation of extracellular matrix (ECM) constituents (e.g., fibronectin, interstitial collagens, hyaluronic acid, and proteoglycans) [29]. Consequently, fibrogenesis is often described as an irrational wound-repairing method [30,31]. Wound healing progresses in four phases: coagulation/clotting, inflammation, fibroblast proliferation/migration, and an ultimate renovating process that restores ordinary tissue structure (Figure 1). Endothelial cells and epithelial cells unleash inflammatory mediators (IMs) in the initial phases of tissue injury, triggering an antifibrinolytic-coagulation mechanism that induces clotting and the formation of a tentative ECM [1]. Platelet accumulation and eventual degranulation facilitate dilation, the penetrability of blood vessels, and the effective recruiting of inflammatory cells (e.g., eosinophils, macrophages, neutrophils, and lymphocytes) to the site of injury [7]. Although neutrophils are the most plentiful inflammatory cells during the initial phases of injury repair, they are rapidly substituted by macrophages following neutrophil degranulation. Enabled macrophages and neutrophils disinfect the injury and remove any interfering species throughout the initial leukocyte migrating cycle [32,33]. Additionally, they comprise a combination of chemokines and cytokines, which enhance the inflammatory mechanism and stimulate fibroblast proliferation and mobilization. Myofibroblasts are assembled from several sources, including fibrocytes, local mesenchymal cells, and the endothelial mesenchymal transition (EMT) pathway [34,35]. Nevertheless, the relative significance of each fibroblast population remains unknown. If stimulated, fibroblasts differentiate into myofibroblasts that convey α-smooth muscle actin (α-SMA) and release ECM elements [30,32]. Ultimately, throughout the injury growth and development period, myofibroblasts stimulate injury contraction, a mechanism through which the wound’s edges move toward the middle, and the epithelial/endothelial cells differentiate and expand through the temporary matrix to rebuild the weakened tissue [1,36]. Fibrosis occurs when an injury is aggressive, when an irritant remains that damages the tissue, or when the healing mechanism is disrupted [1].

Even though several manifestations of PF can be reliably established and investigated in rodents, including particulate matter (e.g., silica and asbestos), drugs (e.g., bleomycin), bronchiolitis obliterans, radiation, and persistent graft-versus-host–induced PF, it remains uncertain whether all of the experimental models genuinely replicate the idiopathic aspect of the disease [37]. On the contrary, several significant advancements have been made using mouse models, with transgenic and knockout mice characterized by improved or diminished sensitivity to PF. Although approximately two decades of investigations have established that TGF-α performs a critical function in the pathogenesis of PF by activating, proliferating, and differentiating collagen-producing myofibroblasts and epithelial cells, no advancement has been reported in translating TGF-α cascade antagonists from the platform to the bedside [38,39].

Based on the available evidence, it appears that the mediators involved in inflammation may be critical for both the initiation and the development of PF [40,41]. IL-17A has been linked to the pathogenesis of PF [42,43,44]. IL-17A levels are elevated in the bronchoalveolar lavage (BAL) of convalescents with IPF. The presence of IL-17A is consistent with chronic neutrophilia in a host of lung diseases, including cystic fibrosis and bacterial pneumonia [44,45,46]. Bleomycin (BLM)-induced IL-17A formation is also strongly focused on TGF-β1 sensing, and recombinant IL-17A-induced fibrosis is based on TGF-β1-downstream profibrotic involvement, implying that IL-17A and TGF-β1 perform a cooperative feature in the progression of PF [44]. Moreover, IL-13 has been commonly attributed to several systemic inflammatory disorders and to fibrosis progression [47]. IL-33 is a powerful stimulator of cutaneous fibrosis induced by IL-13 [48]. This study addresses some of these groundbreaking findings and demonstrates how these novel targets and therapeutic strategies could be used to combat this increasingly complex and heterogeneous disease.

## 3. Soluble Immune Modulators in Pulmonary Fibrosis

Fibrosis develops during the dysregulated wound healing process through the differentiation and proliferation of fibroblasts to pro-fibrotic and apoptosis-resistant myofibroblasts [49]. The highly active myofibroblasts are further affected by various cytokines, chemokines, and growth factors. The involvement of diverse immune cells, e.g., neutrophils, phagocytes, fibrocytes, and T cells, as well as soluble mediators, e.g., cytokines and chemokines, indicates their roles in pulmonary fibrosis [32].

### 3.1. Transforming Growth Factor-Beta-1 (TGF-β1)

TGF-β1 is most commonly involved in the pathogenesis of PF [50]. Various studies on the TGF-β1-based PF progression mechanisms include the activation of the ERK, MAPK, and phosphatidylinositol 3-kinase/Akt pathways [51,52]. TGF-β1 also induces the transcription of pro-collagen I and II via the activation of the serine/threonine kinase and Smad2/3 pathways [53]. Moreover, TGF-β1 promotes the proliferation of fibroblasts via vascular cell adhesion molecules 1 [54] and the differentiation of fibroblasts to myofibroblasts through the induction of α-SMA [55] and galectin-3 [56]. Furthermore, glycogen synthase kinase-3 mediates the induction of TGF-β1 differentiated myofibroblasts [57]. Several therapeutic approaches are underway targeting TGF-β1 to treat PF. Monoclonal antibodies targeting TGF-β1 [58], the inhibition of integrin protein αvβ6 [59], which is an activator of TGF-β1, and the inhibition of the type 1 receptor of TGF-β1 [60] have proven to be effective in alleviating PF. Moreover, the paclitaxel-mediated upregulation of miR-140 results in the downregulation of the TGF-β1/Smad3 pathway and inhibits PF [61].

### 3.2. IL-17

The atypical expression of IL-17 has been linked with several lung disorders, such as pulmonary fibrosis, asthma, and pneumonitis [62]. Recent pieces of evidence support the profibrotic role of IL-17 through the promotion of EMT and the synthesis of collagens via the activation of TGF-β1 [44,63]. However, some conflicting reports show the production of IL-17 in BLM-induced PF by γδ T cells, and mice that are deficient in this T cell subtype show reduced inflammatory responses [64]. The nullification of IL-17-mediated effects is evident in silica-induced PF and results in reduced neutrophil recruitment, Th17 cells, and IL6/IL1β production and an increase in the levels of T-regulatory cells [65]. Although there are deviations in other reports because the source of IL-17 may differ, the IL-17^-/-^ mice were found to be resistant to BLM-induced fibrosis [66]. Moreover, the stimulation of fibroblasts by IL-17 results in the activation of NF-κB, which can be inhibited by inhibiting the IL-17R-associated adaptor protein NF-κB activator 1 (Act1) [67]. The upstream activators of Th17 cells that produce IL-17, such as B cell-activating factor [68], IL-27 [69], osteopontin [70], and adenosine [71], are increased during fibrosis.

### 3.3. IL-1β

Both silica- and BLM-induced PF results in increased NALP3 inflammasome-mediated cytokine IL-1β activity [72] and the overexpression of IL-1β mRNA [73]. Recombinant IL-1β induces PF pathology and the inhibition of the IL-1β receptor attenuated disease pathology [74]. The potent NALP3 inflammasome activators, such as ATP and uric acid, are increased in the BAL fluid of IPF patients and in BLM-induced PF [75]. IL-1β is also well known to collaborate with IL-17, as validated in the fibrosis caused by the inhibition of IL-1β signaling [76,77].

### 3.4. IL-13

IL-13 is produced from diverse origins, notably Th2 lymphocytes, alternatively activated macrophages variant M2a, mast cells, eosinophils, and basophils [78]. Increased IL-13 was found in PF animal models, and the inhibition of IL-13 resulted in reduced fibrotic processes. IL-13-mediated fibrotic events have been initiated via the differentiation of fibroblasts and the release of profibrotic cytokines, e.g., TGF-β, PDGF, and connective tissue growth factor collagen-1 [79,80]. Increased levels of IL-13 [81] and the overexpression of IL-4 and IL-13 receptors [82] were reported in samples obtained from IPF patients. Notably, the signaling downstream of IL-13 is mediated through IL-4Rα, IL-13Rα1, and IL-13Rα2 [83]. The implementation of antibody-based therapy against IL-13 (Anti-IL-13) in treating PF revealed that a potent candidate, tralokinumab, reduced matrix accumulation, lung apoptosis, and lung fibrosis. Tralokinumab also reduced histological, circulating, and lung-associated fibrotic biomarkers in the SCID mouse model [84].

### 3.5. PDGF

PDGF plays a crucial role in the proliferation and differentiation of lung fibroblasts. Overexpression of PDGF has been observed in the macrophages and epithelial cells of clinical IPF lung tissue. Therapeutic approaches made with imatinib, a PDGF-tyrosine kinase inhibitor, aiming to inhibit PDGFR, c-KIT, and Bcr-Abl, significantly attenuated fibrosis in both BLM- and radiation-induced PF [85]. However, despite promising results in preclinical studies, imatinib failed to pass clinical trials [86]. A potent inhibitor of PDGFR named nintedanib has been introduced and has obtained approval from the FDA, along with pirfenidone to treat IPF [87].

### 3.6. IL-6, -8, and -37

Recent clinical data have shown increased IL-6 and IL-8 in acute exacerbated-IPF patients compared to IPF patients. However, the IL-4, IL-10, and IL-13 TGF-β levels are not significantly altered in different disease outcomes [88]. Treatment with the anti-inflammatory cytokine IL-37 attenuated BLM-induced PF by inhibiting collagen deposition and inflammatory infiltration into the lungs. IL-37 treatment reduced MCP-1, IL-6, and TNF-α expression and induced IFN-γ expression in lung tissue [89].

### 3.7. CCL2, 17, 18, and CXCL12

The clinical data demonstrated an increased CCL2 level in the serum and BAL fluid of IPF patients [90]. CCR2-knockout mice show reduced ECM deposition and matrix metalloproteinase (MMP)-2 and -9 production. The IL-10-dependent activation of M2 macrophages, ERK1/2-mediated IL-6 production, and IL-6/STAT signaling-dependent inhibition of the apoptosis of fibroblasts are stimulated by CCL2 [32]. Both CCL17 and its receptor CCR4 are overexpressed in both murine models and IPF patients [91]. The neutralization of CCL17 is beneficial in attenuating pulmonary fibrosis. Increased levels of CCL18 were identified in the BAL fluid, sputum, and serum of IPF patients [92]. The role of CCL18 in disease progression is evident through the increased collagen synthesis from fibroblasts [93]. CXCL12 and its receptor CXCR4 induce the differentiation of fibroblasts to myofibroblasts through the action of Rac1/ERK and JNK signaling and the induction of activator protein-1 [94]. The neutralization of CXCL12 and CXCR4 antagonism attenuates fibrotic collagen deposition and murine PF [95,96].

## 4. Potential Therapeutic Targets of PF

**Figure 2:**
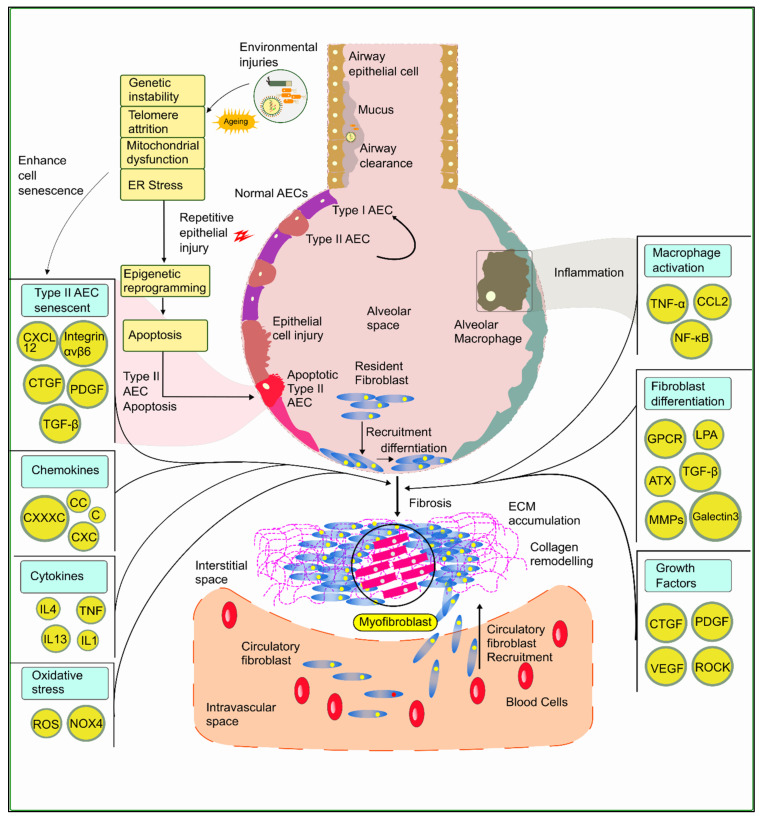
**Potential therapeutic targets aiming at the mechanism of IPF.** Environmental micro-injuries, genetic and epigenetic effects, and thus microbial activities influence the activation pathways and factors that are the inducers of fibrosis and myofibroblast formation. The complex interrelation among key signaling molecules and factors is depicted in this figure, indicating the potential targets for the treatment of IPF identified over years of research.

An array of therapeutic targets has been identified for the treatment, diagnosis, and prognosis of PF. The most notable include cytokines, chemokine, growth factors, cell signaling mediators, collagen remodeling systems, and transcription factors. The potential therapeutic targets of PF are illustrated in Figure 2.

### 4.1. Targeted Antioxidative Pathways

Oxidative stress is one of the main factors linked with the pathogenesis of IPF, and reactive oxygen species (ROS)-generating NADPH oxidase (NOX) enzymes are the main inducers. Several NOX isoforms are liable to fibrotic tissue events. The mentioned NOX isoforms are NOX1 [97,98,99,100], NOX2, and NOX4 [101]. In IPF, the release of cytokines, e.g., TNF-α, is elevated due to the oxidative environment. This induces inducible nitric oxide synthase (iNOS), which is liable to nitric oxide (NO) synthesis and plays a vital role in the pathogenesis of IPF [102,103].

### 4.2. Targeted Cell Signaling Pathways

The PI3K/Akt/mTOR pathway plays a crucial role in regulating metabolism, cell proliferation, and survival. The evidence shows that fibroblast proliferation and differentiation, regulated by the action of TGF-β, can be reduced by inhibiting the PI3K/Akt pathway [104,105]. Omipalisib (GSK2126458), used in the advanced level solid tumor treatment, has been evaluated in clinical trials for IPF, targeting the PI3K/Akt/mTOR pathway [106]. G-protein coupled receptors (GPCRs) are implicated in the disease progression of IPF by activating profibrotic fibroblast promotion [107]. GPR40 and GPR84 are two GPCRs containing free fatty acids, and medium-chain free fatty acids cooperating with long-chain free fatty acids play a crucial role in activating GPR40. In the mouse model, GPR40 genetic deletion expresses the result of kidney fibrosis, whereas GPR84 deletion shows protective effects [108]. Fibroblast recruitment in the BLM model of fibrosis is conducted using lysophosphatidic acid (LPA) via the receptor LPA_1_, which causes an elevation in vascular permeability and subsequent lung injury [109].

Autotaxin, which is elevated in IPF lung tissue, converts lysophospholipids to LPA. This evidence has led to increased attention being paid towards autotaxin as a potential therapeutic target [110,111]. Mice with BLM-induced lung fibrosis show a decreased level of fibroblast recruitment and vascular leakage when the genetic deletion of LPA1 is implemented [108,109,112].

Rho-associated coiled-coil-forming protein kinase (RhoA/ROCK) is a member of the serine-threonine kinase family that plays a vital role in the wound healing process to reform cytoskeletal elements through actomyosin contraction and actin assembly [113]. Fibrotic lesions from mouse models and IPF patients show an increased level of ROCK activity. At the injury site, this induces the profibrotic activation of fibroblasts and epithelial and endothelial cells [114,115]. Clinical studies of the use of fasudil to inhibit ROCK showed that it reduces the number of immune cells in BAL fluid [116,117]. According to Knipe et al., regarding haploinsufficiency studies, both ROCK1 and ROCK2 provide a shield against BLM-associated progression [118,119]. As a member of the MAPK family, stimulated by cytokines and other stress stimuli, JNK modulates different kinds of biological processes involved in the progression of tumor and neurodegenerative diseases [120]. An elevated level of phosphorylated JNK has been found in several types of cells, including the AEC, vascular endothelial cells, alveolar macrophages, smooth muscle cells, and lymphocytes of the lungs of IPF patients [121]. An outstanding clinical result regarding JNK inhibition extended the application of CC 930 into phase II clinical trials (NCT01203943) [106].

### 4.3. Targeted Cytokines and Chemokines

An elevated level of interleukin-13 was found in patients with IPF [80]. Furthermore, mouse models investigating the age of fibrotic progression showed the same scenario [122]. Multiple interactions with the macrophage CCL2 and TGF-β and IL-13 show a complex signaling network in the experimental environment both in vitro and in vivo [123,124,125,126,127]. IL-13, also known as a Th2 cell cytokine, ameliorates lung fibrosis in the experimental mouse model [79,128]. The lysyl oxidase (LOX) enzyme group induces fibroblast growth by cross-linking type 1 collagen molecules via deamination between lysyl and hydroxylysine residues. An elevated level of LOX ligand (LOXL)-2 expression indicates premature senescence [129,130] and the promotion of cell proliferation [129,131]. The application of a monoclonal antibody AB0024 inhibits LOXL-2 and decreases the levels of activated fibroblasts and downregulates the TGF-β signaling pathway [132,133].

### 4.4. Targeted EMT Pathways

Epithelial cells turn into cell types with mesenchymal properties, thus modifying their shape, increasing their motility, and leading to the emergence of mesenchymal markers, e.g., N-cadherin (CDH2), vimentin, and α-SMA [134]. This transition of epithelial cells to mesenchymal properties leads to the progression of myofibroblast formation and is thus another key player in IPF pathogenesis [135]. Wnt signaling is crucial in pulmonary fibrosis as research data show that applying Wnt1-inducible signaling pathway protein-1 neutralizing antibodies to BLM-treated mice attenuates the progression of IPF [136]. Wnt signaling could be a possible crucial therapeutic target for IPF. Dysregulation of MMPs stimulates fibroblast proliferation and extends the event of ECM accumulation and consequently becomes an essential inducer of pulmonary fibrosis development [134]. MMP inhibition could be a retrospective target for the disease management of IPF [135].

### 4.5. Targeted Growth Factors

TGF-β induces epithelial cells, endothelial cells, and mesenchymal cells to influence fibroblast proliferation, chemotaxis, and ECM deposition. Connective tissue growth factor (CTGF) acts as a potent inducer of TGF-β, and an elevated level of CTGF has been found in the BAL fluid of IPF patients [137]. The application of anti-CTGF antibodies in a murine BLM-induced fibrosis model showed a promising reduction in the pathological signs of fibrosis [138]. Another phase II trial study of FG-3019, a human anti-CTGF antibody, is underway to determine the safety and efficacy of this antibody in IPF patients (clinicaltrials.gov identifier NCT01890265) [133]. Aberrant expression of PDGF, VEGF, and FGF, also known as tyrosine kinase receptor ligands, is found in IPF patients [139]. In IPF pathogenesis, PDGF induces the secretion of ECM components and growth factors in fibroblasts [7]. Therefore, PDGF-dependent profibrotic activity is expressed by TGFβ1, FGF, and TNFα [140,141,142]. PDGF also maintains VEGF expression, and this evidence shows that CTGF, PDGF, VEGF, FGF, and TGFβ1 are the most crucial inducers of IPF [143,144], and recent therapeutic developments have aimed at these due to their critical roles in the pathogenesis of IPF.

### 4.6. Targeted Transcription Factors

A conserved DNA-binding domain known as the forkhead box (FoxOs) is part of a transcriptional regulator family consisting of four isoforms, FoxO1, FoxO3, FoxO4, and FoxO6 in mammals [103]. FoxO3 plays a vital role in myofibroblast formation from fibroblasts by suppressing phenotypic changes, where the stimulation of FoxO3 by UCN-01 can turn back the phenotypic change and halt the progression of IPF, and this provides evidence that FoxO3 could be a possible novel target in the treatment of IPF [103]. Heat-shock protein (HSP) 90 is involved in the nuclear localization of Smad, which is the regulating factor in the Smad-dependent signaling cascade of TGF-β [145]. As the leading cytokine in the EMT pathway of IPF progression, TGF-β, in association with HSP90, induces a myofibroblastic phenotypic change of the epithelial cell structure and ECM production [146,147].

### 4.7. Others

Pentraxin 2 (PTX-2) is one of the most important members of the pentraxin family. In the responses of the innate immune system, PTX-2 plays a vital role, including the binding, activation, and regulation of monocytes, macrophages, and neutrophils [148]. PTX-2 causes the differentiation of monocytes into regulatory macrophages [149]. A phase 1 trial (NCT01254409) for PRM-151 (recombinant human PTX-2) has been conducted [150]. The expression of TGF-β receptors on the cell surface of amniotic epithelial cells is regulated by galectin-3, and β-galactoside-binding lectin also influences TGF-β-induced lung fibrosis [56]. Galectin-3-knockout BLM-treated mice showed significant changes in fibrosis progression due to a reduction in their lung collagen level and subsequent lung fibrosis. In response to TGF-β, a decreased level of myofibroblast activation, EMT, and collagen I production have also been documented [56]. Cell adhesion to the ECM and adjacent cells is modulated by transmembrane receptors, such as integrin, which play a vital role in activating intracellular signaling by interacting with the cell cytoskeleton [151]. In the fibrotic areas, type I and Type II AECs express integrin αvβ6 in a decisive manner, as estimated through the immunostaining of IPF lung tissues, related to a higher risk of death with an elevated level of αvβ6 [59,152]. Furthermore, a low-dose treatment study on a BLM mouse model showed that applying a monoclonal antibody to αvβ6 has the potentiality to attenuate fibrosis [59,111].

## 5. Methodology

Articles published between January 2015 and December 2020 in English were considered eligible for our study. PubMed, Google Scholar, and the ISI Web of Science were systematically searched to explore the experimental and clinical data based on the therapeutic role of natural products on pulmonary fibrosis. A total of 216 articles were retrieved from the database. Irrelevant, non-English, redundantly reported compounds, and review articles were excluded. Finally, 93 original research articles were reviewed for the successful intervention of experimental and clinical results.

## 6. Natural Products against Pulmonary Fibrosis with Specific Mechanisms of Action

Natural product-based treatment strategies were found to be very promising in treating PF in preclinical and clinical studies. We have reviewed the recently published literature. The natural products are classified into active compounds, most of which are plant secondary metabolites, crude extract of plants, and traditional herbal medicine, consisting of mixtures of different plant products (root, stem, fruits, seeds, etc.).

Secondary metabolites are chemical compounds produced by plants through the metabolic process and are not directly involved in the plants’ growth and reproduction. Secondary metabolites that play essential roles in treating pulmonary fibrosis include alkaloids, aristolactams, oxoaporphines, amides, indoles, ionones, flavonoids, benzenoids, and steroids, as well as different volatile oils, lipophilic diterpene, tannins, essential oils, triterpenoid, phenolic compound, xanthone, etc. Table 1 summarizes the therapeutic potential of isolated natural products used against PF. Moreover, the organic and aqueous extracts of medicinal plants that effectively alleviate PF disease are summarized in Table 2. Traditional Chinese herbal medicines are prepared mainly via decoction procedures that include boiling plant components, e.g., the stem, bark, leaves, roots, etc. Effective decoction formulas are available for treating experimental PF disease (Table 3).

### 6.1. Inhibition of the EMT Pathway

Specific active components have been studied and confirmed to alleviate PF through the inhibition of the transition of epithelial to mesenchymal cells. β-carbolines are alkaloids derived from *Arenaria kansuensis*, used for treatment in BLM-induced PF mice [153]. β-carbolines significantly decrease pulmonary index and inflammatory cell infiltration. Further in vitro studies with TGF-β-induced A549 cells confirmed the inhibition of the nuclear factor-kappa B (NF-κB) and EMT pathways. Celastrol is a triterpenoid collected from the root of *Trpterygium wilfordii*, which was found to be effective in treating BLM-induced rats [155]. Celastrol also regulates the HSP90-mediated inhibition of EMT. Another xanthonoid compound, gambogic acid, derived from *Garcinia hanburyi* reverse EMT, was revealed in association with reduced vimentin and increased cadherin in TGF-β1-stimulated A549 and HPME cells.

Moreover, in vivo treatment with gambogic acid causes reductions in the pathological score, collagen deposition, and the expression of α-SMA, PDGF, and FGF-2 [158]. Plant sterol β-sitosterol effectively suppressed EMT through the inhibition of the TGF-β1/Snail pathway. β-sitosterol decreased ECM accumulation in human alveolar epithelial cells by inhibiting EMT. Moreover, β-sitosterol downregulated Snail, a transcription factor of EMT, and blocked cell migration [164].

Andrographolide is a diterpenoid lactone that effectively reduces the expression of N-cadherin, α-SMA, vimentin, and collagen deposition in silica-induced pulmonary fibrosis mice [166]. A neolignin compound, Honokiol, derived from *Magnolia officinalis,* inhibits the fundamental pathways of EMT and TGF-β/Smad signaling. Honokiol also significantly mitigated the IL-6/CD44/STAT3 axis both in vitro and in vivo [170]. Nimbolide is a terpenoid compound from *Azadirachta indica*, used for treatment against BLM-induced PF mice and TGF-β-stimulated cells to regulate autophagy through attenuation of the EMT pathway. Nimbolide treatment results in the reduced expression of fibrotic and mesenchymal markers and the increased expression of epithelial markers. Nimbolide also regulates autophagy by reducing microtubule-associated protein 1A/1B-light chain 3 and p-62 expression and increasing Beclin-1 expression [181].

Sulforaphane is an isothiocyanate compound derived from cruciferous vegetables. It restores epithelial morphology in vitro (confirmed by the increased expression of the epithelial marker E-cadherin) and inhibits the expression of EMT-related transcription factors (Slug, Snail, and Twist) and decreases BLM-induced fibronectin expression [184]. Emodin (anthraquinone compound) significantly reduces lung distortion, collagen overproduction, cell infiltration, and proinflammatory cytokine expansion. It induces Nrf2 signaling and dampens p-IIκBα, NF-κB, EMT transition, TGF-β1, and the p-Smad2/3 pathway [193]. Galanin is a flavonoid compound that attenuates inflammatory damage and prevents EMT in BLM-induced PF mice [198].

The effective treatment strategies using plant extracts (Table 2) targeting the EMT pathway include β-peltoboykinolic acid. β-peltoboykinolic acid, from the extract of *Astilbe rubra*, inhibits the COL1 and fibronectin. In addition, ethanol extract of the *A. Rubra* whole plant inhibits TGF-β1-induced EMT in A549 Cells. Furthermore, dichloromethane fractions show the most substantial inhibitory effect on TGF-β1-induced EMT. β-peltoboykinolic acid interrupts the activation of the Smad pathway by TGF-β1 [206].

### 6.2. Inhibition of TGF-β Signaling

Among the soluble immune mediators, TGF-β-mediated signaling is most commonly involved in the pathogenesis of PF. Therefore, an array of approaches targeting the inhibition of TGF-β signaling is inevitable. Salvianolic acid B (SAB) is a polyphenol compound derived from *Salviae miltiorrhza.* It downregulates collagen expression and changes the expression of other fibrotic genes in NIH/3T3 fibroblasts. Treatment with SAB (50μg/mL) inhibited this proliferation with no apparent toxicity. TGF-β-sensitized MRC-5 fibroblasts increased the expression of collagen type 1 alpha 1 (COL1A1) and COL1A2 by 240% and 170%, respectively. SAB significantly downregulated the TGF-β-induced expression of COL1A1, COL1A2, and COL3A1. SAB also attenuated the TGF-β- and TNF-α-induced inhibition of E-cadherin expression [159].

Gentiopicroside (GPS) is a secoiridoid glycoside. GPS significantly decreased the levels of inflammatory cytokines, including TNF-α and IL-1β, in BAL fluid and reduced the content of hydroxyproline in the lungs of PF mice. GPS significantly downregulated the expression of TGF-β1 and CTGF in the lungs of PF mice. Moreover, in an in vitro study in TGF-β1-stimulated A549 cells, GPS dose-dependently inhibited the EMT pathway [162]. The phenolic compound zingerone is derived from ginger. It decreases collagen accumulation and TNF-α, IL-1β, and malondialdehyde (MDA) levels. Ginger inhibits TGF-β1 and iNOS expression and increases superoxide dismutase (SOD) and glutathione peroxidase activity in PF-induced rats [175]. The TGF-β/Smad pathway is inhibited by oridonin, a diterpenoid from *Rabdosia rubesecens*. Oridonin inhibits the mRNA and protein expression of α-SMA and COL1A1 in TGFβ-induced MRC-5 cells and in vivo in BLM-induced PF mice [178]. Myricetin dose-dependently inhibits TGF-β1/Smad2,3 signaling and attenuates fibroblast activation and EMT transition [188]. Rutin is a flavonoid compound from the citrus plant that significantly reduces lactate dehydrogenase activity, as well as the total cell, macrophage, and lymphocyte counts in BAL fluid. Rutin-treated PF-mice showed decreased lung MDA, and nitric oxide increased glutathione and SOD. Moreover, the reduced expression of TGF-β1, Col 1, Col III, and α-SMA is associated with reduced collagen deposition and lung hydroxyproline content [191]. An ex vivo study with a precision-cut lung slice model revealed the role of caffeine in inhibiting TGF-β activation in epithelial cells but not in fibroblasts. Caffeine inhibited α-SMA gene expression and reduced fibrosis after a 5-day treatment in the lung slice model [192]. Rhapontin is effective in lowering ECM deposition and reduced the expression of LOX2 and p-Smad2/3 in vitro. Moreover, rhapontin ameliorates BLM-induced PF with low LOX2 and a high level of AMPK. It also reduces collagen deposition and the expression of TGF-β1, α-SMA, and hypoxia-inducing factor (HIF)-α in the lung [194]. Alantolactone and isoalatolactone are sesquiterpene lactones derived from *Inula helenium L*, which also inhibited the TGF-β1/Smad3 signaling pathway and ultimately reduced the myofibroblast activation and ECM deposition [196]. Paeonol was also found to inhibit TGF-β-induced cellular processes in vitro [169].

Arenaria kansuensis ethanol extract (AE) reduced collagen deposition and α-SMA through inhibition of the TGF-β/Smads pathway. AE is also effective in reducing inflammatory infiltration and inflammatory cytokines in the lungs through the NF-κB/p65 pathway. Moreover, AE reduced oxidative stress by inhibiting ROS levels and induced GSH and SOD activities [203]. Ethyl acetate extract of *Salvia miltiorrhiza* (EASM) alleviates oxidative stress by upregulating Nrf2 and concomitantly downregulating Nox4 in the lungs of BLM-treated mice. EASM reduced ROS generation in fibroblasts by stabilizing Nrf2 protein by promoting kelch-like ECH-associated protein 1 (Keap1) degradation. Nrf2 knockdown in the lungs of BLM-treated mice diminished the inhibitory effects of EASM on fibrosis. EASM suppressed myofibroblast activation with reduced ECM deposition [205].

Black tea extract (BTE) from *Camellia sinensis* reduces α-SMA and TGF-β expression and increases IFN-γ expression in BLM-induced PF mice [209]. Grape seed extracts (GSE) are well known for their inhibitory role in ECM deposition. Moreover, GSE reduces pulmonary inflammation, cytokine, and lactate dehydrogenase activity. GSE alleviates MMP-9 and TGF-β1 protein expression in the lungs [212] and *Rhodiola rosea* L. (RRL) increased SOD and GSH and reduced the hydroxyproline levels in BLM-induced PF rats. The upregulated MMP-9 and α-SMA were decreased significantly in a dose-dependent manner in response to RRL. The levels of TGF-β1 and TIMP-1 in lung tissues were also reduced. In addition, RRL treatment lowered TGF-β1, TNF-α, and IL-6 proteins, indicating that RRL has anti-inflammatory properties in rats with BLM-induced lung fibrosis [238].

Xin Jia Xuan Bai Cheng Qi Decoction (XJXBCQ) treatment in vivo significantly improved lung function, inhibited hydroxyproline levels, and increased the levels of Smad7. XJXBCQ induced a dramatic decrease in the expression of lung TGF-β1 protein. XJXBCQ treatment decreased the expression of α-SMA and fibronectin, IL-17A, and IL-25 in TGFβ1-treated MRC-5 cells. XJXBCQ could downregulate the levels of p-Smad2 in TGF-β1-stimulated MRC-5 cells in a dose-dependent manner. XJXBCQ also regulates the Smad signaling pathway, decreasing Smad2 expression and increasing Smad7 expression in terms of mRNA and protein levels [216]. Qing-Xuan Granule (QX) is a children’s cough medicine patented in China. QX decreased Col I and α-SMA in lung tissues via the inhibition of TGF-β1-Smad2/3 signaling, suppressed EMT, and effectively reversed abnormal mRNA expression of MMP-1, TIMP-1, and LOXL-2 in the lung tissues of BLM-induced PF mice [217]. A Chinese medicine extract triptolide (TPL) inhibits the EMT of lung epithelial cells by directly binding with TGF-β. PQ-induced PF resulted in increased vimentin expression but inhibited E-cadherin expression. TPL reversed EMT progression, increased E-cadherin expression, and decreased vimentin expression. TPL bound to TGF-β and inhibited the TGB1/Smad3 pathway [220].

Renshen pingfei decoction (RPFS) reduces lung injury and the fibrosis degree and improves lung function by decreasing hydroxyproline content. RPFS lowers the gene and protein expression of TGF-β1 and Smad3 in lung tissue. It reduces NFκ-B levels in the BAL fluid of rats. It regulates the level of SOD and MDA in the serum of rats by downregulating the TGF-β1/Smad3-mediated intracellular signal transduction pathway [221]. Astragalus injection (AI) significantly prevented BLM-induced α-SMA, TGF-β1, Jagged1, and Notch1 expression, accompanied by alleviation of the collagen deposition and fibrosis [225]. A combination of salvia miltiorrhiza and ligustrazine (SML) dose-dependently ameliorated BLM-induced PF rats through downregulating TNF-α, TGF-β1, and Smad4. It was also proven to be safer than treating subjects with dexamethasone [228]. Yangyin Yiqi, ixture (YYYQ) treatment at medium and high doses significantly reduced TGF-β1, CTGF, and hydroxyproline levels and the mRNA expression of TGF-β1, TβRI, TβRII, Smad3, α-SMA, laminin, and collagen I. In comparison, YYYQ induced the expression of Smad7 and E-cadherin in the BLM group and the prednisolone-treated BLM group. Therefore, it was supposed to be related to suppressing the EMT and TGF-β1/Smad signaling pathways [229]. Total alkaloids from Alstonia Scholaris (L.) R. Br. decreased Krebs von den Lungen-6, lactate dehydrogenase, TGF-β, hydroxyproline, type I collagen, and malonaldehyde levels. This also enhanced the activity of SOD in the serum and lung tissues. Moreover, the alkaloid treatment resulted in decreased TGF-β and MMP-1 expressions [229]. Ophiocordyceps lanpingensis polysaccharides (OLP) alleviated collagen deposition and oxidative stress and decreased macrophage accumulation. The attenuation of macrophage accumulation inhibited the activation of TGF-β1. OLP also suppressed the expression levels of the TNF-α, IL-1β, IL-6, Oncostatin M (OSM), IL-10, and IL-13 genes [231].

PM014 is a herbal formula that is well known for its usage in the treatment of pulmonary disease. PM014 reduces tissue damage, including lesser degrees of intra-alveolar hyaline membrane formation, inflammatory cell infiltration, and thickness of the bronchiolar epithelium. PM014 treatment significantly inhibits immune cell recruitment and collagen deposition in lung tissue. Moreover, PM014 attenuates TGF-β signaling via the Smad and MAPKs pathways [233]. Yifei sanjie formula (YFSJF) treatment alleviated inflammatory injuries and collagen deposition in PF. The levels of hydroxyproline and the expression of TGF-β1, Col-I, and Col-III were significantly decreased. In addition, YFSJF activates autophagy via activating the PI3K/Akt-mTOR pathway to exert anti-PF effects [234].

### 6.3. Anti-Oxidative and Anti-Inflammatory Roles

Certain compounds are effective in treating PF through modulating inflammation and oxidative stress. Coelonin is a dihydrophenanthrene compound that significantly inhibited LPS-induced IL-1β, IL-6, and TNF-α expression. Moreover, NF-κB and the negative regulator phosphatase and tensin homolog on chromosome ten (PTEN) were significantly reduced [154]. Dioscin (Dio) is a steroidal saponin that significantly reduces type 1 collagen deposition in the lungs. Moreover, the mRNA levels of IL-6, Il-1β, and TNF-α in the lungs of silica-induced PF mice were decreased via Dio treatment. Dio also alleviates pulmonary inflammation by reducing macrophage and lymphocyte infiltration into lung tissues [163]. Asiatic acid is a triterpenoid compound from the medicinal plant *Centella asiatica*, with diverse therapeutic effects. Pretreatment with Asiatic acid inhibits PF progression. It also downregulates inflammatory cell infiltration and pro-inflammatory cytokine and TGF-beta expression [165]. Morin is a very potent anti-inflammatory and anti-oxidative flavonoid compound from *Maclura pomifera.* Treatment with morin attenuates the infiltration of inflammatory cells and hydroxyproline content in the lungs [173]. Apigenin, derived from various vegetables, decreased inflammation and oxidative stress. Apigenin reduces hydroxyproline content and inflammatory cell infiltration. It also increases SOD and PPAR-γ expression in the lungs. Moreover, it causes increased E-cadherin and Smad-7 levels in the lungs and decreased NF-κB, MMP-9, vimentin, and TGF-β expression [179].

Salvianolic acid B (SAB) effectively inhibits myofibroblast trans-differentiation and the upregulation of Nrf2. In vitro treatment with SAB reduces ROS production, increases glutathione, and reduces MDA levels. The anti-fibrotic and anti-oxidative roles of SAB were confirmed in BLM-induced PF rats [185]. Moreover, the anti-inflammatory and anti-oxidative roles of SAB were also established in lipopolysaccharide (LPS)-treated fibroblast cells. SAB and sodium tanshinone IIA sulfonate (STS) downregulate the mRNA and protein expression of IL-1β and TNF-α. Moreover, SAB and STS inhibit α-SMA and COL1α mRNA and protein in TGF-β-induced MRC5 cells [180]. Pterostilbene is a polyphenol compound derived from blueberries that protects against oxidative stress, inflammation, and apoptosis by activating Keap-1/Nrf2. Pterostilbene also inhibited caspase-dependent A20/NF-κB and NLRP3 signaling [182].

GHK (Gly-His-Lys) and GHK-Cu are tri-peptides that have been found to be effective in alleviating inflammatory responses in PF mice, confirmed by analyzing BAL fluid. GHK reduces TNF-α, IL-6, myeloperoxidase (MPO) activity, and collagen deposition. The BLM-induced imbalance of MMP-9/TIMP-1 was reversed after GHK treatment. GHK also prevents the EMT pathway via the TGF-β1/Smad 2/3 and insulin growth factor-1 pathways [186,187]. Rosavin is an alcohol glycoside derived from *Rhodiola rosea* that reduces inflammatory cells’ infiltration into BAL fluid and pro-inflammatory cytokines’ expression in lung tissue. It has also been shown to reduce hydroxyproline and MDA contents. Furthermore, it was found to increase the activities of SOD and glutathione peroxidase in lung tissue. The upregulation of Nrf2 and the downregulation of NF-κB p65, TGF-β1, and α-SMA revealed the mechanism of the rosavin-mediated amelioration of fibrosis [200]. The plant flavonoid quercetin, which can be found in diverse plant sources, reduces inflammation via inhibiting the infiltration of inflammatory cells in the lungs [202]. Schisandrin B (SchB)-treated mice exhibited fewer inflammatory changes and fewer collagen fibers than lung tissues from the BLM group. Moreover, SchB decreased hydroxyproline content and TGF-β1 levels but increased SOD and total antioxidant capacity in the lungs [172]. Glaucocalyxin A (GlnA) is a terpenoid compound that significantly reduces collagen deposition and hydroxyproline content in the lungs. Moreover, GlnA inhibits the infiltration of macrophages and neutrophils and attenuates pro-inflammatory cytokine levels in BAL fluid [176]. Co-treatment with quercetin and gallic acid significantly decreases hydroxyproline, TNF-α, and GSH levels and increases catalase and SOD activity in the lungs compared with both single phytochemical-treated groups [199]. Coco et al. studied the ethanolic extract of Baru nuts, a native Brazilian species. The extract comprises phenolic compounds such as gallic acid, potentially exhibiting antioxidative and wound healing activity in human NCI-H441 and A549 lung epithelial cell lines [239].

Date palm sap (DPS) treatment reversed BLM-mediated increased MDA and SOD levels and decreased catalase activity. DPS further decreased hydroxyproline levels and morphological lesions induced by BLM [207]. In rats, *Nigella sativa* oil (NSO) treatment reduces the inflammatory index and fibrosis score and increases the urinary secretion of histidine, fumarate, allantoin, and malate. Therefore, it is assumed that NSO attenuates PF through resistance from lung, kidney, and liver tissues [208]. The nutraceutical role of aged garlic extract (AGE) in alleviating pulmonary fibrosis was validated in a TiO_2_-induced pulmonary and hepatic animal model. AGE diminishes TiO_2_-induced toxicity by downregulating pulmonary MMP-9, TIMP-9, TGF-β1, collagen-1α, and fibronectin mRNA [210]. The methanolic extract of *Myrtus communis* (Myrtle) significantly reduces parenchymal inflammation, hydroxyproline content, and lipid peroxidation. It also increases catalase activity in PF mice [213]. The protective effects of *Berberis vulgaris* fruit extract (BVFE) against paraquat (PQ)-induced PF rats have also been validated. PQ significantly increases the lungs’ MDA, hydroxyproline, TNF-α, IL-6, and TGF-β1 levels. BVFE ameliorated the biochemical and histological lung alterations induced by PQ [214]. *Pistacia lentiscus* oil (PLO) decreases the lipoperoxidation caused by BLM. PLO also protected against BLM-induced lung fibrosis and oxidative stress [215].

Citrus alkaline extracts (CAE) mitigated pulmonary fibrosis in a BLM-induced mouse model by preventing fibroblast senescence. CAE inhibited the expression of the senescent biomarkers P16^INK4a^, P21, and the senescence-associated β-galactosidase (SA-β-Gal) positive cells, as well as the etoposide-induced senescence of lung fibroblasts in vitro. CAE regulates the senescence-associated secretory phenotype by inhibiting senescence in fibroblasts. Further mechanism studies confirmed that CAE inhibits lung fibroblast senescence via a P53-dependent mechanism, and cyclooxygenase-2 activation is required for CAE to inhibit P53-dependent fibroblast senescence [211].

Chuanxiong Kangxian granules (CCKG) attenuate BLM-induced pulmonary fibrosis in rats. CCKG reduces BLM-induced collagen deposition, oxidative stress, and inflammatory responses, reducing the expression of MMP-2 and MMP-9, which are proteins involved in the construction of ECM [219]. Modified Kushen Gancao Formula (mKG) is a Chinese herbal medicine which shows anti-inflammatory activities. mKG treatment significantly decreased pulmonary alveolitis, fibrosis scores, hydroxyprolines, Col-1, Col-3 contents, IL-6, IL-17, and TGF-β in BLM-induced lung tissues [222]. Astragaloside IV (ASV) alleviated collagen deposition and the suppression of EMT in pulmonary fibrosis in vivo. In addition, ASV inhibited TGF-β and activated FOXO3a, which also inhibits TGF-β-induced EMT via the PI3K/Akt pathway [224].

Jinshu Huanxian formula (JHF) increased glutathione, glutathione peroxidase, catalase, and SOD and decreased the content of MPO. JHF also inhibits the expression of NOX4 and induces Nrf2 [230]. Polysaccharides from *Ganoderma luciderma* (PGL) ameliorate PF by increasing glutathione, glutathione peroxidase, catalase, and SOD and decreasing the contents of MDA and hydroxyproline [232]. In addition to the anti-TGF-β activity of PM014, it also acts via the inhibition of the expression of cytokines (IL-6, IL-13, IL-1b, and TGF-β), chemokines (MIP1a, MCP1, and CCL4), and fibrosis-related genes (Col3al and Fn1) in radiation-induced PF mice [235]. In the BLM-induced model, Pyunkanghwan (Pyunkang-tang) extract (PGT) alleviates the characteristic histopathological features of lung fibrosis and inhibits fibrotic lesions. PGT also inhibits BLM-induced MDA, demonstrating its protective effect against lipid peroxidation in lung cell membranes. In addition, PGT decreased TGF-β-stimulated type I collagen synthesis in vitro [236]. However, PGT inhibits TGF-β1-induced collagen accumulation and EMT by inhibiting the PI3K/Akt signaling pathway [237]. Feifukang (FFK) treatment significantly reduced the BLM-induced increase in hydroxyproline content, collagen I, and a-SMA expression. Moreover, FEK regulates JAK-STAT signaling via the phosphorylation of Smad3, STAT3, and JAK1 [218].

### 6.4. Modulation of Cellular Signaling

Magnesium isoglycyrrhizinate (MgIG) treatment reduced collagen deposition, ROS production, and TGF-β1 elevation in vitro. Administration of MgIG achieved lower expression levels of Nox4 and p38/MAPK/Akt in vivo and in vitro [156]. Alpha-mangostin (α-MG) in vivo treatment dramatically reduced the expression of α-SMA and Col 1 at both the mRNA and the protein levels. α-MG treatments also reduced the abnormal expression of TGF-β1 and the phosphorylation of Smad 2/3 in the lungs. Moreover, α-MG alleviates the process of fibrogenesis by promoting AMPK-mediated inhibition of NOX4 expression and TGF-β1-induced trans-differentiation of lung fibroblast [157].

Polydatin (PD) treatment suppressed mycoplasma pneumonia-induced lung injury in mice by inhibiting the expression of inflammatory factors and the development of fibrotic scars. PD also inhibits the activation of the NLRP3 inflammasome and the NF-κB pathway [161]. Zingerone and Tetrandrine-hydroxypropyl-β-cyclodextrin inclusion compound (TET-HP-β-CD) are a ketone and an alkaloid natural compound which have effectively reduced MDA and hydroxyproline content in vivo. Moreover, zingerone inhibits the signaling pathways of NF-κB and MAPKs [167,240]. Juglanin (Jug) also reduced the expression of fibrotic hallmarks, including TGF-β1, fibronectin, MMP-9, α-SMA, and collagen I. The role of Jug in suppressing the stimulator of interferon genes (Sting) was confirmed in TGF-β-incubated cells [168]. Phycocyanin is a phycobilin compound from cyanobacteria that reduces MPO, IL-6, TNF-α, and type 1 alveolar epithelial cells. It also inhibits fibroblast proliferation and attenuates EMT [171]. Parthenolide, derived from *Tanacetum parthenium*, significantly reduced cell viability and migration and inhibited EMT markers in lung epithelial cells. The treatment of parthenolide in vivo results in attenuation of the pathological markers of fibrosis through inhibition of the NFκB/Snail signaling pathway [177].

Coumarin compound wedelolactone (WEL), derived from *Eclipta Prostratacoumarin*, reduces inflammatory cell infiltration and collagen deposition in lung tissues. WEL also impairs the expression of fibrotic markers (α-SMA, Col I), the reduction in anti-fibrotic markers (E-cadherin), and the prevention of BLM-induced TGF-β1 and Smad2/3 phosphorylation [189]. Madecacassoside is a vital ingredient of *Centella asiatica* that significantly alters cellular signaling in the gut. Oral but not i.p. administration of madecacassoside has significant anti-fibrotic effects. Madecacassoside increases hepatocyte growth factor levels in colon tissue through the upregulation of PPAR-γ mRNA, nuclear translocation, and DNA binding activity in madecassoside-treated colonic epithelial cells [190]. Berberine is an alkaloid compound derived from various Chinese herbs that alleviates PF pathology when administered through either oral or rectal routes. Berberine also induces HGF and PTEN mRNA and protein expression via PPAR-γ in the colon of PF-mice [174]. Scutellarein alleviates the differentiation and proliferation of fibroblasts to myofibroblasts. Inhibiting fibroblast differentiation represses TGF-β/Smad signaling. Further in vitro studies have confirmed the inhibition of cell proliferation by repressing PI3K/Akt signaling and the inhibition of apoptosis by Bcl2 associated X protein/Bcl2 signaling [183].

### 6.5. Inhibition of ECM Deposition

The deposition of collagen, a vital ingredient of the ECM, facilitates the pathology of pulmonary fibrosis. Therefore, the targeted inhibition of ECM could be an essential strategy to treat pulmonary fibrosis. The curcuminoid ingredients from curcumin and curcumol reduce ECM deposition via autophagy. In vitro curcumin or curcumol treatment in human lung fibroblast cells significantly reduced hydroxyproline, α-SMA, Col-I, and Col-III deposition. Furthermore, N-terminal pro-peptide for type I collagen (PINP), N-terminal pro-peptide for type III collagen, and prolyl-hydroxylase related to ECM were deregulated in a dose-dependent manner [160].

Hydroxysafflor yellow A (HSYA) is a flavonoid from *Carthamus tinctorius* that reduces collagen deposition in the lung. HYSA also alleviates the BLM-induced increase in the mRNA of TGF-β, α-SMA, and collagen I and downregulates Smad3 phosphorylation [195]. 4-methoxy phenethylamine is a biological amine from the pericarp of *Citrus reticulate,* reducing hydroxyproline in serum and lung tissue. It also downregulates TGF-β expression [197]. Treatment with ascorbic acid in paraquat-induced PF mice significantly reduced immune cell infiltration, the secretion of IL-17 and TGF-β, and ECM deposition. Moreover, vitamin C increases the anti-oxidative enzymes, SOD, and catalase levels [201]. The antioxidant properties of *Radix puerariae* extracts (RPEs) ameliorate PQ-induced PF. RPE significantly suppressed lung fibrosis by downregulating Fstl 1 pathways through decreased miR-21 expression. Moreover, RPE suppressed the expression of CTGF, TGF-β1, p38MAPK, NF-kB65, pSmad2/3, and MMP-9 protein levels and attenuated pulmonary fibrosis [223]. The Chinese herbal medicine Hong Jing Tian contains small RNA (HJT-sRNA-m7) involved in reducing fibrotic markers in vitro and in vivo. The decoction formula contains the phosphocholines PC (18:0/18:2) and PC (16:0/18:2), which facilitate the uptake of small RNAs by living cells. HJT-sRNA-m7 effectively reduces the gene expressions of α-SMA, fibronectin, and collagen type I α 1 (COL1A1) [226].

## 7. Natural Product-Based Randomized Controlled Trials on IPF

Double-blinded randomized controlled trials were performed with 60 patients (male and female, aged 18–75 years). The control group received a matched placebo plus pirfenidone, and the experimental group was co-treated with pirfenidone and maimendong decoction (MD) for 24 weeks. The evaluations were based on both primary and secondary outcomes. The mean change in forced vital capacity and exacerbation at 4, 12, and 24 weeks were the primary outcomes. However, the study also examined changes in the St. Geroge’s Respiratory Questionnaire total score, forced expiratory volume in 1 s percentage/forced vital capacity, diffusing capacity of carbon monoxide, natriuretic peptide, etc. The results confirmed that MD plus pirfenidone significantly improves lung function and quality of life and decreases acute exacerbation [227]. In another randomized controlled trial, 46 patients were recruited with silicosis. Another 18 patients were recruited as a normal control group. Intervention with Yiqi Houxue decoction (YHD) at one dose daily for 14 days and the Western treatment were compared with a symptomatically normal control group. YHD significantly reduced co-stimulatory molecule CD40 protein and mRNA expression compared to the control group [241]. Furthermore, a randomized controlled trial studied one hundred IPF patients who were randomly divided into a control (20) and treatment group (80). The control group was treated with Jinshuibao capsules (JCs), and the treatment group received Feiwei granules (FGs) for six months. The FG-treated group showed greater efficacy in specific parameters than the control group. However, there were no significant differences in most of the parameters and therefore FGs and JCs were similar in terms of IPF treatment [242].

## 8. Summary and Future Directions

Pulmonary fibrosis is the result of architectural deformation of the lungs. Recently, surviving SARS-CoV-2 patients have also potentially suffered from PF-like pathogenesis, as observed in clinical evidence. In this review, we have briefly discussed the mechanisms of PF connected with immune regulation. Moreover, potential therapeutic targets for treating PF are introduced. We have reviewed the recent (2015–2020) experimental and clinical data demonstrating the promising roles of active plant compounds, plant extracts, and traditional herbal medicines in treating PF. Although there are plenty of experimental results on the therapeutic effects of natural products against PF, well-designed prospective cohort studies with large sample sizes and long-term follow-up experiments are inadequate. Target-specific validation of each compound, employing advanced drug discovery strategies, e.g., in silico, high-throughput screening, systems modeling and simulation, etc., are inevitable in order to introduce new effective drugs against PF.

## Figures and Tables

**Table 1:** Isolated active compounds from secondary plant metabolites.

No.	Active Compound	Structure	Categories	Source	Experiment Model	Therapeutic Target	Ref.
1.	β-carbolines	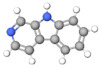	Alkaloid	*Arenaria kansuensis*	In vivo: BLM-induced PF mice; in vitro: A549, RAW264	Inhibiting NF-κB/p65 and EMT transition	[153]
2.	Coelonin	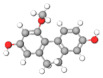	Dihydrophenanthrene	*Bletilla striata*	In vivo: BLM-induced PF rats	Anti-inflammatory and anti-fibrotic	[154]
3.	Celastrol	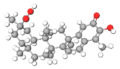	Triterpenoid	*Tripterygium wilfordii* and *Celastrus regelii.*	In vivo: BLM-induced PF rats; in vitro: A549 cells	TGF-β1/Smad2/3-mediated inhibition of EMT	[155]
4.	Magnesium isoglycyrrhizinate (MgIG)	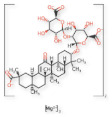	Triterpenoid saponin glycoside	*Glycyrrhiza glabra*	In vivo: radiation-induced PF mice; in vitro: human fetal lung fibroblasts-1 (HFL1)	Inhibiting fibroblast differentiation via p38MAPK/Akt/Nox4 pathway	[156]
5.	Alpha-mangostin (α-MG)	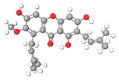	Xanthone	Mangosteen (*Garcinia mangostana)*	In vivo: BLM-induced PF mice; in vitro: mouse primary lung fibroblasts (PLFs)	Inhibiting AMP-activated protein kinase	[157]
6.	Gambogic acid	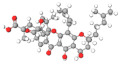	Xanthonoid	*Garcinia hanburyi*	In vivo: BLM-induced PF rats. In vitro: HLF-1, HPMECs, and A549 cells	Reversing EMT and suppress TGF-β1/Smad3 pathway	[158]
7.	Salvianolic acid B (SAB)	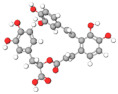	Polyphenol	*Salviae miltiorrhiza*	In vivo: BLM-induced PF mice; in vitro: NIH/3T3 fibroblasts, MRC-5 fibroblasts, A549 cells	Inhibiting TGF-β signaling	[159]
8.	Curcumin and curcumol	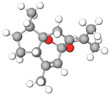	Curcuminoid	Rhizomes of *Curcuma zedoaria*	in vitro: human lung fibroblast (HLF)	Inhibiting collagen deposition via autophagy mechanism	[160]
9.	Polydatin (PD)	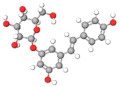	Resveratrol glucoside	*Polygonum cuspidatum*	In vivo: MP-induced PF mice; in vitro: BEAS-2B cells	NLRP3 inflammasome and NF-κB pathway	[161]
10.	Gentiopicroside(GPS)	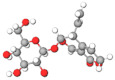	Secoiridoid glycoside	*Gentianascabra, Gentiana lutea*	In vivo: BLM-induced PF mice; in vitro: A549 cells	Anti-inflammatory and anti-fibrotic via TGF-β-1	[162]
11.	Dioscin (Dio)	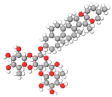	Steroidal saponin	Dioscorea nipponica Makino	In vivo: silica-induced PF mice; in vitro: RAW264.7 cell line, NIH-3T3 cell line	Inhibiting pro-inflammatory cytokines through ASK-1-p38/JNK signaling	[163]
12.	β-sitosterol	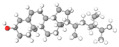	Phytosterols	*stigma and style of maize (Zea mays)*	In vitro: human lung adenocarcinoma epithelial cells, A549.	Suppression of EMT via the TGF-β1/Snail pathway	[164]
13.	Asiatic Acid	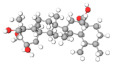	Triterpenoid	*Centella Asiatica*	In vivo: BLM-induced mice	Anti-inflammatory and anti-fibrotic	[165]
14.	Andrographolide	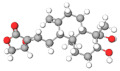	labdane diterpenoid lactone	*Andrographis paniculata*	In vivo: silica-induced pulmonary fibrosis mice	Anti-inflammatory and EMT transition	[166]
15.	zingerone (vanillylacetone)	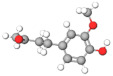	Ketone	*Zingiber officinale*	In vivo: BLM-induced PF rats	Inhibiting NF-κB and MAPKs	[167]
16.	Tetrandrine (TET-HP-β-CD)	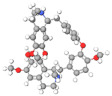	Alkaloid	*Stephania tetrandra, S. Moore*	In vivo: BLM-induced PF rats	Alleviating inflammation and fibrosis	[168]
17.	Juglanin	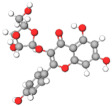	Flavonoid	*Polygonum aviculare*	In vivo: BLM-induced PF mice	Inhibition of stimulator of interferon genes (Sting) signaling	[168]
18.	Paeonol	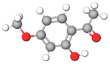	Phenols	*Paeonia suffruticosa*	In vivo: BLM-induced PF mice	Inhibition of MAPKs/Smad3 signaling	[169]
19.	Honokiol	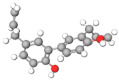	Neolignan	*Magnolia officinalis*	In vivo: BLM-induced PF mice	Inhibiting EMT and TGF-β/Smad signaling both in vitro and in vivo	[170]
20.	Phycocyanin	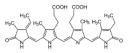	Phycobilins	Cyanobacteria	In vivo: BLM-induced PF mice	Attenuating PF via TLR2-MyD88-NFκB signaling	[171]
21.	Schisandrin B	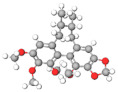	Tanin	*Schisandra chinensis*	In vitro: A549 cells. BLM-induced PF mice	Attenuating BLM-induced PF via wingless/integrase-1 signaling	[172]
22.	Morin	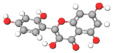	Flavonoid	*Maclura pomifera*	In vivo: BLM-induced PF mice	Anti-oxidative and anti-inflammatory	[173]
23.	Berberine	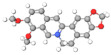	Alkaloid	Chinese herbs	In vivo: BLM-induced PF mice	Activation of PPAR-γ. Expression of HGF in the colon.	[174]
24.	Zingerone	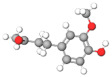	Phenolics, Ketone	*Zingiber officinale*	In vivo: BLM-induced PF rats	Modulating the expression of TGF-β1 and iNOS	[175]
25.	Glaucocalyxin A	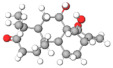	Diterpenoid	*Rabdosia japonica*	In vivo: BLM-induced PF mice	Antagonism of leukocyte infiltration. Proinflammatory cytokine production.	[176]
26.	Parthenolide	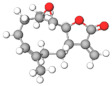	Sesquiterpene	*Tanacetum parthenium*	In vivo: BLM-induced PF mice; in vitro: primary lung fibroblast, HFL1 cells, A549 cells	Attenuating BLM-induced PF via NF-κB/Snail signaling	[177]
27.	Oridonin	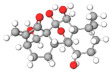	Diterpenoid	*Rabdosia rubesecens*	In vitro: MRC5 cells, in vivo: BLM-induced PF mice	Regulating TGFβ/Smad pathway	[178]
28.	Apigenin	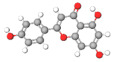	Flavonoid	Vegetables	In vivo: BLM-induced PF mice	Anti-oxidative and PPARγ expression	[179]
29.	Salvianolic acid B and sodium tanshinone IIA	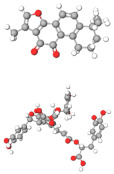	Phenolics	*Salvia miltiorrhiza*	In vitro: MR5 cells	Anti-inflammation and anti-fibrotic	[180]
30.	Nimbolide	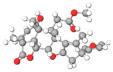	Terpenoids	*Azadirachta indica*	In vivo: BLM-induced PF mice; in vitro: TGF-beta induced cell line	Autophagy regulator through attenuation of EMT pathway	[181]
31.	Perostilbene	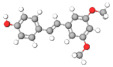	Polyphenol	Blueberries	In vivo: LPS-induced PF mice	Preventing fibrosis by suppressing oxidative stress, inflammation, and apoptosis	[182]
32.	Scutellarein	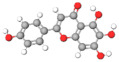	Flavonoid	*Scutellaria Lateriflora*	In vivo: BLM-induced PF mice; in vitro: human pulmonary fibroblast	Affecting fibroblast differentiation, proliferation, and apoptosis	[183]
33.	Sulforaphane	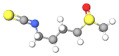	Isothiocyanates	Cruciferous vegetables	In vivo: BLM-induced PF mice; in vitro: A549 cell, MRC-5 cell	Inhibiting EMT transition	[184]
34.	Salvianolic acid B	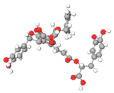	Phenolic acids	*Salvia miltiorrhiza*	In vivo: BLM-induced PF rats; in vitro: MRC-5 cells	Inhibiting myofibroblast trans-differentiation via upregulation of Nrf2	[185]
35.	GHK peptide	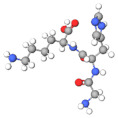	Tripeptide	Plasma protein	In vivo: BLM-induced PF mice	Anti-oxidative and anti-inflammation	[186]
36.	Glycyl-L-histidyl-l-lysine (GHK)-Cu	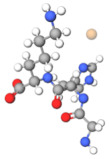	Tripeptide	Plasma protein	In vivo: BLM-induced PF mice	Anti-oxidative and anti-inflammation	[187]
37.	Myricetin	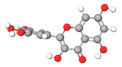	Flavonoid	*Commercially purchase*	In vivo: BLM-induced PF mice; in vitro: human, mouse pulmonary epithelial cell, lung fibroblast cell	Inhibiting TGF-β1 via targeting HSP90β	[188]
38.	Wedelolactone	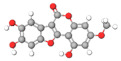	Coumarin	*Eclipta prostrata*	In vivo: BLM-induced PF mice	Activation of AMPK and regulating Raf-MAPK pathway	[189]
39.	Madecacassoside	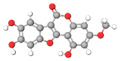	Pentacyclic triterpenoid	*Centella asiatica*	In vivo: BLM-induced PF mice	Promoting hepatocyte growth factor in the colon via PPAR-γ	[190]
40.	Rutin	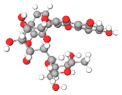	Flavonoid	Citrus plant	In vivo: BLM-induced PF mice	Inhibiting TGF-β1-α/SMA/Col I and III pathway	[191]
41.	Caffeine	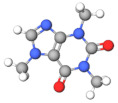	Alkaloid	Tea, coffee, cacao, etc.	ex vivo: precision-cut lung slice model; in vitro: epithelial and lung fibroblast cells	Inhibiting TGF-β activation	[192]
42.	Emodin	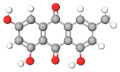	Anthraquinone	Rhubarb	In vivo: BLM-induced PF rats; in vitro: alveolar epithelial cell	Inhibiting EMT transition, TGF-β1, p-Smad2/3	[193]
43.	Rhapontin	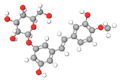	Stilbene glycosides	Rheum genus/ Rhubarb	In vivo: BLM-induced PF mice; in vitro: primary lung fibroblast; LP-stimulated human THP-1	Reducing collagen deposition, TGF-beta1, α-SMA, HIF-α expression	[194]
44.	Hydroxysafflor yellow A (HSYA)	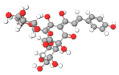	Flavonoid	*Carthamus tinctorius*	In vivo: BLM-induced PF mice; in vitro: A549 cells	Reducing ECM deposition	[195]
45.	Alantolactone and isoalatolactone	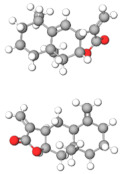	Sesquiterpene lactone	*Inula helenium L*	In vivo: BLM-induced PF mice; in vitro: TGF-β1-induced human lung fibroblasts.	Inhibiting TGF-β1/Smad3 signaling pathway	[196]
46.	4-methoxyphenethylamine	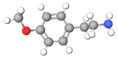	Biological amine	Pericarp of *Citrus Reticulata*	In vivo: BLM-induced PF rats model; in vitro: human embryonic lung fibroblast	Inhibiting TGF-β1	[197]
47.	Galangin	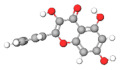	Flavonoid	Galangal	In vivo: BLM-induced PF mice	Attenuating EMT and inflammatory damage	[198]
48.	Quercetin and Gallic acid	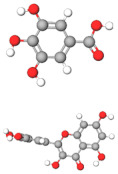	Flavonoid	Quercetin and Gallic acid	In vivo: BLM-induced PF rats	Anti-oxidative and anti-inflammatory	[199]
49.	Rosavin	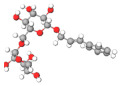	Alcohol glycosides	*Rhodiola rosea*	In vivo: BLM-induced PF mice	Anti-fibrotic and anti-inflammatory	[200]
50.	Ascorbic Acid	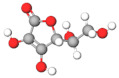	Vitamin	Fruits	In vivo: PQ-induced PF	Reducing IL-6, IL-17a, TGF-beta	[201]
51.	Quercetin	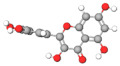	Flavonoid	Diverse plant sources	Amiodarone intra-tracheal instillation	Antioxidant and anti-inflammatory	[202]

The molecular structures of active compounds were generated from the online repository www.molview.org, accessed on 14 October 2021. Here, each color in molecules denotes each atom. Therefore, gray = carbon, white= hydrogen, red=oxygen, blue=nitrogen, and yellow=sulfur.

**Table 2:** Plant extract-based therapeutic approaches against pulmonary fibrosis.

No.	Formula	Source and Components	Experiment Model	Therapeutic Targets	Ref.
1.	Arenaria kansuensis	Chinese herbal (plant extracts)	In vivo: PQ-induced PF mice	Activation of Nrf2 pathway and the inhibition of NF-kb/TGF-beta1/Smad2/3 pathway.	[203]
2.	Tanshinone IIa	*Salvia miltiorrhiza* (Danshen) (plant extracts)	In vivo: silica-induced PF rats; in vitro: A549 and HBE cells.	Inhibition of EMT	[204]
3.	Ethyl acetate extract of Salvia miltiorrhiza (EASM)	Dried roots and rhizomes of *Salvia miltiorrhiza* (plant extracts)	In vivo: BLM-induced PF mice; in vitro: mouse embryo fibroblasts (NIH-3T3)	Inhibition of TGF-β1/Smad3 signaling by downregulating protein kinase C delta	[205]
4.	Β-peltoboykinolic acid	*Astilbe rubra* (plant extracts)	In vitro: A549 cells	Inhibition of EMT	[206]
5.	Date palm sap (DPS)	*Date palm Phoenix dactylifera* L. (plant extracts)	In vivo: BLM-induced PF rats	Reversing inflammation and oxidative stress	[207]
6.	Nigella sativa oil (NSO)	*Nigella sativa* (plant extracts)	In vivo: BLM-induced PF rats	Anti-inflammatory	[208]
7.	Black tea extract (BTE)	*Camellia sinensis* (plant extracts)	In vivo: BLM-induced PF mice	Anti-fibrotic	[209]
8.	Aged garlic extract (AGE)	*Alluim sativum* (plant extracts)	In vivo: TiO_2_-induced toxicity	Attenuating hepatic inflammation and pulmonary fibrosis	[210]
9.	Citrus alkaline extracts (CAE)	Pericarp of *Citrus reticulata* (flavanone, alkaloid)	In vivo: BLM-induced PF mice; in vitro: primary murine lung fibroblasts	Preventing fibroblast senescence via activation of cyc-2	[211]
10.	Grape seed extracts (GSEs)	Flavonoids, ascorbic acid (vitamin C), tocopherols, citric acid, limonoids, sterols, and minerals	In vivo: BLM-induced PF mice	Inhibition of MMP-9 and TGF-β1	[212]
11.	Myrtle	*Myrtus communis* L. (flavonoids, tannins, and essential oils)	In vivo: BLM-induced PF rats	Anti-inflammation and anti-oxidative	[213]
12.	Berberis vulgaris fruit extract (BVFE)	*Berberis vulgaris* (phenolic and flavonoids compounds)	In vivo: PQ-induced PF rats	Antioxidant and anti-inflammatory	[214]
13.	Pistacia lentiscus oil	*Pistacia lentiscus* (phenolic compounds and fatty acids)	In vivo: BLM-induced PF rats	Antioxidant	[215]
14.	Mixture of extracts	The roots and rhizomes of *Rhodiola rosea* L. (RRL) (phenylpropanoids, organic acids, and flavonoids)	In vivo: BLM-induced rats	TGF-β1 signaling transduction in lung tissues	[216]

**Table 3:** Traditional herbal medicine.

No.	Name	Components	Experiment Model	Therapeutic Targets	Ref.
1.	Xin jia xuan bai cheng qi decoction (XJXBCQ)	Gypsum fibrosum, Rhei radix et rhizome, Semen armeniacae amarum, Trichosanthis pericarpium, Persicae semen, Eupolyphaga steleophaga (in 10:3:2:5:3:3 ratio)	In vivo: BLM-induced PF rats; in vitro: MRC-5 cells	TGF-β1-Smad2/3 signaling	[216]
2.	Qing-xuan granule (QX)	Mulberry leaves, mint, bitter apricot kernels (fried), Platycodonis Radix, Paeoniae Radix Alba, Citri Reticulatae Pericaricarpium, Aurantii Fructus, Armeniacae Semen Amarum, Glycyrrhizae Radix et Rhizoma, and Mori Folium.	In vivo: BLM-induced PF mice; in vitro: MLE-12 cells	Downregulating TGF-β1-Smad2/3 signaling	[217]
3.	Feifukang (FFK)	Astragalus membranaceus (Fisch) Bge., Codonopsis pilosula (Franch.) Nannf., Ophiopogon japonicus, Schisandra chinensis, Panax notoginseng (Burk.) F. H. Chen., Bulbus fritillariae thunbergii, Rhizoma anemarrhenae, and Glycyrrhiza uralensis	In vivo: BLM-induced PF mice	Inhibition of JAK-STAT signaling pathway	[218]
4.	Chuanxiong Kangxian granules (CCKG)	Combination of Chuanxiong and Kangxian	In vivo: BLM-induced PF rats	Inhibition of oxidative stress and inflammation	[219]
5.	Triptolide (TPL)	Tripterygium wilfordii	In vivo: PQ-induced PF mice	Binds with TGF-β and inhibits Smad3, E-cadherin, and Vimentin.	[220]
6.	Renshen pingfei decoction (RPFS)	Panax ginseng, Glycyrrhiza uralensis, Morus alba, Lycium chinense, Asparagus cochinchinensis, Citrus reticulata, Anemarrhena asphodeloides.	In vivo: BLM-induced PF rats	Downregulating TGF-β1/Smad3 signaling pathway.	[221]
7.	Modified Kushen Gancao Formula (mKG)	Roots of *Angelica sinensis*, roots of *Sophora flavescens*, rhizome of *Glycyrrhiza uralensis*.	In vivo: BLM-induced PF mice	Anti-inflammatory and alleviation of hydroxyproline	[222]
8.	Radix puerariae extracts (RPES)	Root of *Pueraria lobata* (Wild)	In vivo: PQ-induced PF mice	Fstl pathways and oxidative stress by inhibiting mir-21 expression	[223]
9.	Astragaloside IV (ASV)	*Astragalus membranaceus*	In vivo: BLM-induced PF rats; in vitro: A549 cells	Antioxidative stress and antiinflammation and inhibit EMT	[224]
10.	Astragalus injection (AI)	Astragaloside	In vivo: BLM-induced PF rats	Modulating the TGF-b/Smad pathway in rats	[225]
11.	Hong jing tian (HGT)	*Rhodiola crenulate*	In vivo: BLM-induced PF mice. In vitro: MRC-5, A549, HEK293T, NCI-N87 cell lines	Effectively reducing the expression of fibrotic hallmark genes and proteins	[226]
12.	Maimendong decoction (MD)	Radix Ophiopogonis japonici (Maimendong) 10 g, Pinellia ternate (Banxia) 15 g, Radix Ginseng (Renshen) 5 g, Polygonum cuspidatum (Huzhang) 10 g, Ligusticum (Chuanxiong) 10 g, Salviae Miltiorrhizae Radix Et Rhizoma (Danshen) 15 g and licorice (Gancao) 5 g.	Human	Forced vital capacity, times of acute exacerbation.	[227]
13.	Salvia miltiorrhiza and ligustrazine (SML)		In vivo: BLM-induced PF rats	Modulating the expression of TNF-α and TGF-β1.	[228]
14.	Yangyin Yiqi mixture (YYYQ)	Traditional Chinese medicine	In vivo: BLM-induced PF rats	Suppressing TGF-*β*1/Smad signal pathway and EMT	[229]
15.	Jinshu huanxian formula (JHF)	Chinese traditional medicine	In vivo: BLM-induced PF rats	The antioxidative mechanism via Nrf2 upregulation	[230]
16.	*Ophiocordyceps lanpingensis* polysaccharides (OLP)	Ophiocordyceps lanpingensis	In vivo: BLM-induced PF mice	Reducing the accumulation of macrophages	[231]
17.	Polysachharides from Ganoderma luciderma (PGL)	Ganoderma luciderma	In vivo: BLM-induced PF rats	Reducing pulmonary index, inflammatory cell infiltration, collagen deposition	[232]
18.	PM014	Chung-Sang-Bo-Ha-Tang	In vivo: BLM-induced PF mice	Inhibiting TGF-β1 signaling via Smad-dependent and MAPK pathways	[233]
19.	Yifei Sanjie formula (YFSJF)	Astragalus, Atractylodes macrocephala Koidz, Saposhnikovia, Fritillaria thunbergii, Semen Sinapis, Curcuma zedoaria rhizomes, Panax notoginseng	In vivo: BLM-induced PF mice; In vitro: human lung fibroblasts	Inhibiting the expression of TGF-β1	[234]
20.	PM014	Modified herbal formuladerived from Chung-Sang-Bo-Ha-Tang (CSBHT)	In vivo: Radiation-induced mice	Inhibiting the expression of cytokines, chemokines, and fibrosis-related genes.	[235]
21.	Pyunkanghwan (Pyunkang-tang) extract (PGT)	Water extracts of six herbs	In vivo: BLM-induced PF rats; in vitro: MLG 2908 cells	Inhibition of lipid peroxidation	[236]
22.	Buyang huanwu tang(BHT)	Astragalus membranaceus (60 g), Radix Paeoniae Rubra (18 g), Rhizoma Ligustici Wallichii (9 g), Angelica sinensis (18 g),Pheretima aspergillum (9 g), Amygdalus persica (9 g), and Carthamus tinctorius (9 g)	In vitro: A549 cells	Inhibition of EMT via inhibiting TGF-β1	[237]

## Data Availability

Not applicable.

## References

[1] Thannickal V.J., Toews G.B., White E.S., Lynch J.P., Martinez F.J. (2004). Mechanisms of pulmonary fibrosis. Annu. Rev. Med..

[2] Smith M.L. (2016). Update on Pulmonary Fibrosis: Not All Fibrosis Is Created Equally. Arch. Pathol. Lab. Med..

[3] Richeldi L., Collard H.R., Jones M.G. (2017). Idiopathic pulmonary fibrosis. Lancet.

[4] Wakwaya Y., Brown K.K. (2019). Idiopathic Pulmonary Fibrosis: Epidemiology, Diagnosis and Outcomes. Am. J. Med. Sci..

[5] King T.E., Pardo A., Selman M. (2011). Idiopathic pulmonary fibrosis. Lancet.

[6] Meyer K.C. (2017). Pulmonary fibrosis, part I: Epidemiology, pathogenesis, and diagnosis. Expert Rev. Respir. Med..

[7] Martinez F.J., Collard H.R., Pardo A., Raghu G., Richeldi L., Selman M., Swigris J.J., Taniguchi H., Wells A.U. (2017). Idiopathic pulmonary fibrosis. Nat. Rev. Dis. Primers.

[8] Wolff D., Reichenberger F., Steiner B., Kahl C., Leithauser M., Skibbe T., Friedrich T., Terpe H., Helbig W., Freund M. (2002). Progressive interstitial fibrosis of the lung in sclerodermoid chronic graft-versus-host disease. Bone Marrow Transplant..

[9] George P.M., Wells A.U., Jenkins R.G. (2020). Pulmonary fibrosis and COVID-19: The potential role for antifibrotic therapy. Lancet. Respir. Med..

[10] Vasarmidi E., Tsitoura E., Spandidos D.A., Tzanakis N., Antoniou K.M. (2020). Pulmonary fibrosis in the aftermath of the COVID-19 era (Review). Exp. Ther. Med..

[11] Ojo A.S., Balogun S.A., Williams O.T., Ojo O.S. (2020). Pulmonary Fibrosis in COVID-19 Survivors: Predictive Factors and Risk Reduction Strategies. Pulm. Med..

[12] Lechowicz K., Drozdzal S., Machaj F., Rosik J., Szostak B., Zegan-Baranska M., Biernawska J., Dabrowski W., Rotter I., Kotfis K. (2020). COVID-19: The Potential Treatment of Pulmonary Fibrosis Associated with SARS-CoV-2 Infection. J. Clin. Med..

[13] Kayhan S., Kocakoc E. (2020). Pulmonary Fibrosis Due to COVID-19 Pneumonia. Korean J. Radiol..

[14] Wang L., Li S., Yao Y., Yin W., Ye T. (2021). The role of natural products in the prevention and treatment of pulmonary fibrosis: A review. Food Funct..

[15] Meyer K.C., Danoff S.K., Lancaster L.H., Nathan S.D. (2015). Management of idiopathic pulmonary fibrosis in the elderly patient: Addressing key questions. Chest.

[16] Ryu J.H., Moua T., Daniels C.E., Hartman T.E., Yi E.S., Utz J.P., Limper A.H. (2014). Idiopathic pulmonary fibrosis: Evolving concepts. Mayo Clin. Proc..

[17] Ryerson C.J., Cottin V., Brown K.K., Collard H.R. (2015). Acute exacerbation of idiopathic pulmonary fibrosis: Shifting the paradigm. Eur. Respir. J..

[18] Collard H.R., Moore B.B., Flaherty K.R., Brown K.K., Kaner R.J., King T.E., Lasky J.A., Loyd J.E., Noth I., Olman M.A. (2007). Acute exacerbations of idiopathic pulmonary fibrosis. Am. J. Respir. Crit. Care Med..

[19] Song J.W., Hong S.B., Lim C.M., Koh Y., Kim D.S. (2011). Acute exacerbation of idiopathic pulmonary fibrosis: Incidence, risk factors and outcome. Eur. Respir. J..

[20] Sgalla G., Biffi A., Richeldi L. (2016). Idiopathic pulmonary fibrosis: Diagnosis, epidemiology and natural history. Respirology.

[21] Moore B.B., Moore T.A. (2015). Viruses in idiopathic pulmonary fibrosis etiology and exacerbation. Ann. Am. Thorac. Soc..

[22] Naik P.K., Moore B.B. (2010). Viral infection and aging as cofactors for the development of pulmonary fibrosis. Expert Rev. Respir. Med..

[23] He Y., Thummuri D., Zheng G., Okunieff P., Citrin D.E., Vujaskovic Z., Zhou D. (2019). Cellular senescence and radiation-induced pulmonary fibrosis. Transl. Res..

[24] Huang Y., Zhang W., Yu F., Gao F. (2017). The cellular and molecular mechanism of radiation-induced lung injury. Med. Sci. Monit..

[25] Gogali A., Wells A.U. (2010). New pharmacological strategies for the treatment of pulmonary fibrosis. Ther. Adv. Respir. Dis..

[26] Sathiyamoorthy G., Sehgal S., Ashton R.W. (2017). Pirfenidone and Nintedanib for Treatment of Idiopathic Pulmonary Fibrosis. South. Med. J..

[27] Rogliani P., Calzetta L., Cavalli F., Matera M.G., Cazzola M. (2016). Pirfenidone, nintedanib and N-acetylcysteine for the treatment of idiopathic pulmonary fibrosis: A systematic review and meta-analysis. Pulm. Pharmacol. Ther..

[28] Young A., Koduri G., Batley M., Kulinskaya E., Gough A., Norton S., Dixey J. (2007). Mortality in rheumatoid arthritis. Increased in the early course of disease, in ischaemic heart disease and in pulmonary fibrosis. Rheumatology.

[29] Upagupta C., Shimbori C., Alsilmi R., Kolb M. (2018). Matrix abnormalities in pulmonary fibrosis. Eur. Respir. Rev..

[30] Pardo A., Selman M. (2016). Lung fibroblasts, aging, and idiopathic pulmonary fibrosis. Ann. Am. Thorac. Soc..

[31] Wolters P.J., Collard H.R., Jones K.D. (2014). Pathogenesis of idiopathic pulmonary fibrosis. Annu. Rev. Pathol. Mech. Dis..

[32] Kolahian S., Fernandez I.E., Eickelberg O., Hartl D. (2016). Immune mechanisms in pulmonary fibrosis. Am. J. Respir. Cell Mol. Biol..

[33] Malaviya R., Kipen H.M., Businaro R., Laskin J.D., Laskin D.L. (2020). Pulmonary toxicants and fibrosis: Innate and adaptive immune mechanisms. Toxicol. Appl. Pharmacol..

[34] Noble P.W., Barkauskas C.E., Jiang D. (2012). Pulmonary fibrosis: Patterns and perpetrators. J. Clin. Investig..

[35] Luppi F., Kalluri M., Faverio P., Kreuter M., Ferrara G. (2021). Idiopathic pulmonary fibrosis beyond the lung: Understanding disease mechanisms to improve diagnosis and management. Respir. Res..

[36] Ding Q., Luckhardt T., Hecker L., Zhou Y., Liu G., Antony V.B., DeAndrade J., Thannickal V.J. (2011). New insights into the pathogenesis and treatment of idiopathic pulmonary fibrosis. Drugs.

[37] Moore B.B., Murray L., Das A., Wilke C.A., Herrygers A.B., Toews G.B. (2006). The role of CCL12 in the recruitment of fibrocytes and lung fibrosis. Am. J. Respir. Cell Mol. Biol..

[38] Border W.A., Noble N.A. (1994). Transformation growth factor β in tissue fibrosis. New Engl. J. Med..

[39] Kisseleva T., Brenner D.A. (2008). Mechanisms of fibrogenesis. Exp. Biol. Med..

[40] Heukels P., Moor C.C., von der Thüsen J.H., Wijsenbeek M.S., Kool M. (2019). Inflammation and immunity in IPF pathogenesis and treatment. Respir. Med..

[41] Bringardner B.D., Baran C.P., Eubank T.D., Marsh C.B. (2008). The role of inflammation in the pathogenesis of idiopathic pulmonary fibrosis. Antioxid. Redox Signal..

[42] Celada L.J., Kropski J.A., Herazo-Maya J.D., Luo W., Creecy A., Abad A.T., Chioma O.S., Lee G., Hassell N.E., Shaginurova G.I. (2018). PD-1 up-regulation on CD4+ T cells promotes pulmonary fibrosis through STAT3-mediated IL-17A and TGF-β1 production. Sci. Transl. Med..

[43] Zhang J., Wang D., Wang L., Wang S., Roden A.C., Zhao H., Li X., Prakash Y.S., Matteson E.L., Tschumperlin D.J. (2019). Profibrotic effect of IL-17A and elevated IL-17RA in idiopathic pulmonary fibrosis and rheumatoid arthritis-associated lung disease support a direct role for IL-17A/IL-17RA in human fibrotic interstitial lung disease. Am. J. Physiol. Lung Cell Mol. Physiol..

[44] Wilson M.S., Madala S.K., Ramalingam T.R., Gochuico B.R., Rosas I.O., Cheever A.W., Wynn T.A. (2010). Bleomycin and IL-1β-mediated pulmonary fibrosis is IL-17A dependent. J. Exp. Med..

[45] Brodlie M., McKean M.C., Johnson G.E., Anderson A.E., Hilkens C.M.U., Fisher A.J., Corris P.A., Lordan J.L., Ward C. (2011). Raised interleukin-17 is immunolocalised to neutrophils in cystic fibrosis lung disease. Eur. Respir. J..

[46] Hsu E., Shi H., Jordan R.M., Lyons-Weiler J., Pilewski J.M., Feghali-Bostwick C.A. (2011). Lung tissues in patients with systemic sclerosis have gene expression patterns unique to pulmonary fibrosis and pulmonary hypertension. Arthritis Rheum..

[47] Wynn T.A. (2004). Fibrotic disease and the TH1/TH2 paradigm. Nat. Rev. Immunol..

[48] Rankin A.L., Mumm J.B., Murphy E., Turner S., Yu N., McClanahan T.K., Bourne P.A., Pierce R.H., Kastelein R., Pflanz S. (2010). IL-33 Induces IL-13–Dependent Cutaneous Fibrosis. J. Immunol..

[49] Fernandez I.E., Eickelberg O. (2012). New cellular and molecular mechanisms of lung injury and fibrosis in idiopathic pulmonary fibrosis. Lancet.

[50] Ask K., Bonniaud P., Maass K., Eickelberg O., Margetts P.J., Warburton D., Groffen J., Gauldie J., Kolb M. (2008). Progressive pulmonary fibrosis is mediated by TGF-β isoform 1 but not TGF-β3. Int. J. Biochem. Cell Biol..

[51] Hayashida T., Decaestecker M., Schnaper H.W. (2003). Cross-talk between ERK MAP kinase and Smad signaling pathways enhances TGF-β-dependent responses in human mesangial cells. FASEB J. Off. Publ. Fed. Am. Soc. Exp. Biol..

[52] Asano Y., Ihn H., Yamane K., Jinnin M., Mimura Y., Tamaki K. (2004). Phosphatidylinositol 3-kinase is involved in α2(I) collagen gene expression in normal and scleroderma fibroblasts. J. Immunol..

[53] Roberts A.B., Russo A., Felici A., Flanders K.C. (2003). Smad3: A key player in pathogenetic mechanisms dependent on TGF-β. Ann. N. Y. Acad. Sci..

[54] Agassandian M., Tedrow J.R., Sembrat J., Kass D.J., Zhang Y., Goncharova E.A., Kaminski N., Mallampalli R.K., Vuga L.J. (2015). VCAM-1 is a TGF-β1 inducible gene upregulated in idiopathic pulmonary fibrosis. Cell. Signal..

[55] Gauldie J., Sime P.J., Xing Z., Marr B., Tremblay G.M. (1999). Transforming growth factor-β gene transfer to the lung induces myofibroblast presence and pulmonary fibrosis. Curr. Top. Pathology. Ergeb. Pathol..

[56] Mackinnon A.C., Gibbons M.A., Farnworth S.L., Leffler H., Nilsson U.J., Delaine T., Simpson A.J., Forbes S.J., Hirani N., Gauldie J. (2012). Regulation of transforming growth factor-β1-driven lung fibrosis by galectin-3. Am. J. Respir. Crit. Care Med..

[57] Baarsma H.A., Engelbertink L.H., van Hees L.J., Menzen M.H., Meurs H., Timens W., Postma D.S., Kerstjens H.A., Gosens R. (2013). Glycogen synthase kinase-3 (GSK-3) regulates TGF-β(1)-induced differentiation of pulmonary fibroblasts. Br. J. Pharmacol..

[58] Wang Q., Wang Y., Hyde D.M., Gotwals P.J., Koteliansky V.E., Ryan S.T., Giri S.N. (1999). Reduction of bleomycin induced lung fibrosis by transforming growth factor β soluble receptor in hamsters. Thorax.

[59] Horan G.S., Wood S., Ona V., Li D.J., Lukashev M.E., Weinreb P.H., Simon K.J., Hahm K., Allaire N.E., Rinaldi N.J. (2008). Partial inhibition of integrin α(v)β6 prevents pulmonary fibrosis without exacerbating inflammation. Am. J. Respir. Crit. Care Med..

[60] Higashiyama H., Yoshimoto D., Kaise T., Matsubara S., Fujiwara M., Kikkawa H., Asano S., Kinoshita M. (2007). Inhibition of activin receptor-like kinase 5 attenuates bleomycin-induced pulmonary fibrosis. Exp. Mol. Pathol..

[61] Wang C., Song X., Li Y., Han F., Gao S., Wang X., Xie S., Lv C. (2013). Low-dose paclitaxel ameliorates pulmonary fibrosis by suppressing TGF-β1/Smad3 pathway via miR-140 upregulation. PLoS ONE.

[62] Gurczynski S.J., Moore B.B. (2018). IL-17 in the lung: The good, the bad, and the ugly. Am. J. Physiol. Lung Cell Mol. Physiol..

[63] Mi S., Li Z., Yang H.Z., Liu H., Wang J.P., Ma Y.G., Wang X.X., Liu H.Z., Sun W., Hu Z.W. (2011). Blocking IL-17A promotes the resolution of pulmonary inflammation and fibrosis via TGF-β1-dependent and -independent mechanisms. J. Immunol..

[64] Braun R.K., Ferrick C., Neubauer P., Sjoding M., Sterner-Kock A., Kock M., Putney L., Ferrick D.A., Hyde D.M., Love R.B. (2008). IL-17 producing gammadelta T cells are required for a controlled inflammatory response after bleomycin-induced lung injury. Inflammation.

[65] Chen Y., Li C., Weng D., Song L., Tang W., Dai W., Yu Y., Liu F., Zhao M., Lu C. (2014). Neutralization of interleukin-17A delays progression of silica-induced lung inflammation and fibrosis in C57BL/6 mice. Toxicol. Appl. Pharmacol..

[66] Zhou X., Loomis-King H., Gurczynski S.J., Wilke C.A., Konopka K.E., Ptaschinski C., Coomes S.M., Iwakura Y., van Dyk L.F., Lukacs N.W. (2016). Bone marrow transplantation alters lung antigen-presenting cells to promote TH17 response and the development of pneumonitis and fibrosis following gammaherpesvirus infection. Mucosal Immunol..

[67] Dong Z., Yang Y., Zhang T., Li Y., Kang Q., Lei W., Cao Y., Niu X., Wang D., Tai W. (2013). siRNA-Act1 inhibits the function of IL-17 on lung fibroblasts via the NF-kappaB pathway. Respir. Int. Rev. Thorac. Dis..

[68] Francois A., Gombault A., Villeret B., Alsaleh G., Fanny M., Gasse P., Adam S.M., Crestani B., Sibilia J., Schneider P. (2015). B cell activating factor is central to bleomycin- and IL-17-mediated experimental pulmonary fibrosis. J. Autoimmun..

[69] Dong Z., Lu X., Yang Y., Zhang T., Li Y., Chai Y., Lei W., Li C., Ai L., Tai W. (2015). IL-27 alleviates the bleomycin-induced pulmonary fibrosis by regulating the Th17 cell differentiation. BMC Pulm. Med..

[70] Oh K., Seo M.W., Kim Y.W., Lee D.S. (2015). Osteopontin Potentiates Pulmonary Inflammation and Fibrosis by Modulating IL-17/IFN-gamma-secreting T-cell Ratios in Bleomycin-treated Mice. Immune Netw..

[71] Luo F., Le N.B., Mills T., Chen N.Y., Karmouty-Quintana H., Molina J.G., Davies J., Philip K., Volcik K.A., Liu H. (2016). Extracellular adenosine levels are associated with the progression and exacerbation of pulmonary fibrosis. FASEB J. Off. Publ. Fed. Am. Soc. Exp. Biol..

[72] Cassel S.L., Eisenbarth S.C., Iyer S.S., Sadler J.J., Colegio O.R., Tephly L.A., Carter A.B., Rothman P.B., Flavell R.A., Sutterwala F.S. (2008). The Nalp3 inflammasome is essential for the development of silicosis. Proc. Natl. Acad. Sci. USA.

[73] Cavarra E., Carraro F., Fineschi S., Naldini A., Bartalesi B., Pucci A., Lungarella G. (2004). Early response to bleomycin is characterized by different cytokine and cytokine receptor profiles in lungs. Am. J. Physiol. Lung Cell Mol. Physiol..

[74] Gasse P., Mary C., Guenon I., Noulin N., Charron S., Schnyder-Candrian S., Schnyder B., Akira S., Quesniaux V.F., Lagente V. (2007). IL-1R1/MyD88 signaling and the inflammasome are essential in pulmonary inflammation and fibrosis in mice. J. Clin. Investig..

[75] Riteau N., Gasse P., Fauconnier L., Gombault A., Couegnat M., Fick L., Kanellopoulos J., Quesniaux V.F., Marchand-Adam S., Crestani B. (2010). Extracellular ATP is a danger signal activating P2X7 receptor in lung inflammation and fibrosis. Am. J. Respir. Crit. Care Med..

[76] Nalbant A., Eskier D. (2016). Genes associated with T helper 17 cell differentiation and function. Front. Biosci..

[77] Song L., Weng D., Dai W., Tang W., Chen S., Li C., Chen Y., Liu F., Chen J. (2014). Th17 can regulate silica-induced lung inflammation through an IL-1β-dependent mechanism. J. Cell. Mol. Med..

[78] Passalacqua G., Mincarini M., Colombo D., Troisi G., Ferrari M., Bagnasco D., Balbi F., Riccio A., Canonica G.W. (2017). IL-13 and idiopathic pulmonary fibrosis: Possible links and new therapeutic strategies. Pulm. Pharmacol. Ther..

[79] Belperio J.A., Dy M., Burdick M.D., Xue Y.Y., Li K., Elias J.A., Keane M.P. (2002). Interaction of IL-13 and C10 in the pathogenesis of bleomycin-induced pulmonary fibrosis. Am. J. Respir. Cell Mol. Biol..

[80] Hancock A., Armstrong L., Gama R., Millar A. (1998). Production of interleukin 13 by alveolar macrophages from normal and fibrotic lung. Am. J. Respir. Cell Mol. Biol..

[81] Jakubzick C., Choi E.S., Carpenter K.J., Kunkel S.L., Evanoff H., Martinez F.J., Flaherty K.R., Toews G.B., Colby T.V., Travis W.D. (2004). Human pulmonary fibroblasts exhibit altered interleukin-4 and interleukin-13 receptor subunit expression in idiopathic interstitial pneumonia. Am. J. Pathol..

[82] Park S.W., Ahn M.H., Jang H.K., Jang A.S., Kim D.J., Koh E.S., Park J.S., Uh S.T., Kim Y.H., Park J.S. (2009). Interleukin-13 and its receptors in idiopathic interstitial pneumonia: Clinical implications for lung function. J. Korean Med. Sci..

[83] Hilton D.J., Zhang J.G., Metcalf D., Alexander W.S., Nicola N.A., Willson T.A. (1996). Cloning and characterization of a binding subunit of the interleukin 13 receptor that is also a component of the interleukin 4 receptor. Proc. Natl. Acad. Sci. USA.

[84] Murray L.A., Zhang H., Oak S.R., Coelho A.L., Herath A., Flaherty K.R., Lee J., Bell M., Knight D.A., Martinez F.J. (2014). Targeting interleukin-13 with tralokinumab attenuates lung fibrosis and epithelial damage in a humanized SCID idiopathic pulmonary fibrosis model. Am. J. Respir. Cell Mol. Biol..

[85] Aono Y., Nishioka Y., Inayama M., Ugai M., Kishi J., Uehara H., Izumi K., Sone S. (2005). Imatinib as a novel antifibrotic agent in bleomycin-induced pulmonary fibrosis in mice. Am. J. Respir. Crit. Care Med..

[86] Daniels C.E., Lasky J.A., Limper A.H., Mieras K., Gabor E., Schroeder D.R., Imatinib I.P.F.S.I. (2010). Imatinib treatment for idiopathic pulmonary fibrosis: Randomized placebo-controlled trial results. Am. J. Respir. Crit. Care Med..

[87] Richeldi L., du Bois R.M., Raghu G., Azuma A., Brown K.K., Costabel U., Cottin V., Flaherty K.R., Hansell D.M., Inoue Y. (2014). Efficacy and safety of nintedanib in idiopathic pulmonary fibrosis. N. Engl. J. Med..

[88] Papiris S.A., Tomos I.P., Karakatsani A., Spathis A., Korbila I., Analitis A., Kolilekas L., Kagouridis K., Loukides S., Karakitsos P. (2018). High levels of IL-6 and IL-8 characterize early-on idiopathic pulmonary fibrosis acute exacerbations. Cytokine.

[89] Li Y., Gao Q., Xu K., Peng X., Yuan X., Jiang W., Li M. (2018). Interleukin-37 Attenuates Bleomycin-Induced Pulmonary Inflammation and Fibrosis in Mice. Inflammation.

[90] Suga M., Iyonaga K., Ichiyasu H., Saita N., Yamasaki H., Ando M. (1999). Clinical significance of MCP-1 levels in BALF and serum in patients with interstitial lung diseases. Eur. Respir. J..

[91] Yogo Y., Fujishima S., Inoue T., Saito F., Shiomi T., Yamaguchi K., Ishizaka A. (2009). Macrophage derived chemokine (CCL22), thymus and activation-regulated chemokine (CCL17), and CCR4 in idiopathic pulmonary fibrosis. Respir Res..

[92] Prasse A., Probst C., Bargagli E., Zissel G., Toews G.B., Flaherty K.R., Olschewski M., Rottoli P., Muller-Quernheim J. (2009). Serum CC-chemokine ligand 18 concentration predicts outcome in idiopathic pulmonary fibrosis. Am. J. Respir. Crit. Care Med..

[93] Atamas S.P., Luzina I.G., Choi J., Tsymbalyuk N., Carbonetti N.H., Singh I.S., Trojanowska M., Jimenez S.A., White B. (2003). Pulmonary and activation-regulated chemokine stimulates collagen production in lung fibroblasts. Am. J. Respir. Cell Mol. Biol..

[94] Lin C.H., Shih C.H., Tseng C.C., Yu C.C., Tsai Y.J., Bien M.Y., Chen B.C. (2014). CXCL12 induces connective tissue growth factor expression in human lung fibroblasts through the Rac1/ERK, JNK, and AP-1 pathways. PLoS ONE.

[95] Phillips R.J., Burdick M.D., Hong K., Lutz M.A., Murray L.A., Xue Y.Y., Belperio J.A., Keane M.P., Strieter R.M. (2004). Circulating fibrocytes traffic to the lungs in response to CXCL12 and mediate fibrosis. J. Clin. Investig..

[96] Shu H.K., Yoon Y., Hong S., Xu K., Gao H., Hao C., Torres-Gonzalez E., Nayra C., Rojas M., Shim H. (2013). Inhibition of the CXCL12/CXCR4-axis as preventive therapy for radiation-induced pulmonary fibrosis. PLoS ONE.

[97] Akasaki T., Ohya Y., Kuroda J., Eto K., Abe I., Sumimoto H., Iida M. (2006). Increased expression of gp91phox homologues of NAD(P)H oxidase in the aortic media during chronic hypertension: Involvement of the renin-angiotensin system. Hypertens. Res..

[98] Masamune A., Watanabe T., Kikuta K., Satoh K., Shimosegawa T. (2007). NADPH oxidase plays a crucial role in the activation of pancreatic stellate cells. Am. J. Physiol. Gastrointest. Liver Physiol..

[99] Stas S., Whaley-Connell A., Habibi J., Appesh L., Hayden M.R., Karuparthi P.R., Qazi M., Morris E.M., Cooper S.A., Link C.D. (2007). Mineralocorticoid receptor blockade attenuates chronic overexpression of the renin-angiotensin-aldosterone system stimulation of reduced nicotinamide adenine dinucleotide phosphate oxidase and cardiac remodeling. Endocrinology.

[100] Wang P., Tang F., Li R., Zhang H., Chen S., Liu P., Huang H. (2007). Contribution of different Nox homologues to cardiac remodeling in two-kidney two-clip renovascular hypertensive rats: Effect of valsartan. Pharmacol. Res..

[101] Hecker L., Cheng J., Thannickal V.J. (2012). Targeting NOX enzymes in pulmonary fibrosis. Cell. Mol. Life Sci..

[102] Zhu B., Ma A.Q., Yang L., Dang X.M. (2013). Atorvastatin attenuates bleomycin-induced pulmonary fibrosis via suppressing iNOS expression and the CTGF (CCN2)/ERK signaling pathway. Int. J. Mol. Sci..

[103] Altintas N., Erboga M., Aktas C., Bilir B., Aydin M., Sengul A., Ates Z., Topcu B., Gurel A. (2016). Protective Effect of Infliximab, a Tumor Necrosis Factor-Alfa Inhibitor, on Bleomycin-Induced Lung Fibrosis in Rats. Inflammation.

[104] Conte E., Fruciano M., Fagone E., Gili E., Caraci F., Iemmolo M., Crimi N., Vancheri C. (2011). Inhibition of PI3K Prevents the Proliferation and Differentiation of Human Lung Fibroblasts into Myofibroblasts: The Role of Class I P110 Isoforms. PLoS ONE.

[105] Conte E., Gili E., Fruciano M., Korfei M., Fagone E., Iemmolo M., Lo Furno D., Giuffrida R., Crimi N., Guenther A. (2013). PI3K p110$γ$ overexpression in idiopathic pulmonary fibrosis lung tissue and fibroblast cells: In vitro effects of its inhibition. Lab. Investig..

[106] Lv M., Liu Y., Ma S., Yu Z. (2019). Current advances in idiopathic pulmonary fibrosis: The pathogenesis, therapeutic strategies and candidate molecules. Future Med. Chem..

[107] Haak A.J., Ducharme M.T., Diaz Espinosa A.M., Tschumperlin D.J. (2020). Targeting GPCR Signaling for Idiopathic Pulmonary Fibrosis Therapies.

[108] Sato S., Yanagihara T., Kolb M.R.J. (2019). Therapeutic targets and early stage clinical trials for pulmonary fibrosis. Expert Opin. Investig. Drugs.

[109] Tager A.M., LaCamera P., Shea B.S., Campanella G.S., Selman M., Zhao Z., Polosukhin V., Wain J., Karimi-Shah B.A., Kim N.D. (2008). The lysophosphatidic acid receptor LPA1 links pulmonary fibrosis to lung injury by mediating fibroblast recruitment and vascular leak. Nat. Med..

[110] Oikonomou N., Mouratis M.A., Tzouvelekis A., Kaffe E., Valavanis C., Vilaras G., Karameris A., Prestwich G.D., Bouros D., Aidinis V. (2012). Pulmonary autotaxin expression contributes to the pathogenesis of pulmonary fibrosis. Am. J. Respir. Cell Mol. Biol..

[111] Hewitt R.J., Maher T.M. (2019). Idiopathic Pulmonary Fibrosis: New and Emerging Treatment Options. Drugs Aging.

[112] Kolb M., Bonella F., Wollin L. (2017). Therapeutic targets in idiopathic pulmonary fibrosis. Respir. Med..

[113] Julian L., Olson M.F. (2014). Rho-associated coiled-coil containing kinases (ROCK), structure, regulation, and functions. Small GTPases.

[114] Shimizu Y., Dobashi K., Sano T., Yamada M. (2014). Rock activation in lung of idiopathic pulmonary fibrosis with oxidative stress. Int. J. Immunopathol. Pharmacol..

[115] Zhou Y., Huang X., Hecker L., Kurundkar D., Kurundkar A., Liu H., Jin T.H., Desai L., Bernard K., Thannickal V.J. (2013). Inhibition of mechanosensitive signaling in myofibroblasts ameliorates experimental pulmonary fibrosis. J. Clin. Investig..

[116] Jiang C., Huang H., Liu J., Wang Y., Lu Z., Xu Z. (2012). Fasudil, a Rho-kinase inhibitor, attenuates bleomycin-induced pulmonary fibrosis in mice. Int. J. Mol. Sci..

[117] Bei Y., Hua-Huy T.Ô., Duong-Quy S., Nguyen V.H., Chen W., Nicco C., Batteux F., Dinh-Xuan A.T. (2013). Long-term treatment with fasudil improves bleomycin-induced pulmonary fibrosis and pulmonary hypertension via inhibition of Smad2/3 phosphorylation. Pulm. Pharmacol. Ther..

[118] Knipe R.S., Probst C.K., Lagares D., Franklin A., Spinney J.J., Brazee P.L., Grasberger P., Zhang L., Black K.E., Sakai N. (2018). The rho kinase isoforms ROCK1 and ROCK2 each contribute to the development of experimental pulmonary fibrosis. Am. J. Respir. Cell Mol. Biol..

[119] Sontake V., Gajjala P.R., Kasam R.K., Madala S.K. (2019). New therapeutics based on emerging concepts in pulmonary fibrosis. Expert Opin. Ther. Targets.

[120] Davies C., Tournier C. (2012). Exploring the function of the JNK (c-Jun N-terminal kinase) signalling pathway in physiological and pathological processes to design novel therapeutic strategies. Biochem. Soc. Trans..

[121] Yoshida K., Kuwano K., Hagimoto N., Watanabe K., Matsuba T., Fujita M., Inoshima I., Hara N. (2002). MAP kinase activation and apoptosis in lung tissues from patients with idiopathic pulmonary fibrosis. J. Pathol..

[122] Cieslik K.A., Taffet G.E., Carlson S., Hermosillo J., Trial J.A., Entman M.L. (2011). Immune-inflammatory dysregulation modulates the incidence of progressive fibrosis and diastolic stiffness in the aging heart. J. Mol. Cell. Cardiol..

[123] Chun Geun L., Homer R.J., Zhu Z., Lanone S., Wang X., Koteliansky V., Shipley J.M., Gotwals P., Noble P., Chen Q. (2001). Interleukin-13 induces tissue fibrosis by selectively stimulating and activating transforming growth factor β_1_. J. Exp. Med..

[124] Lee J.H., Kaminski N., Dolganov G., Grunig G., Koth L., Solomon C., Erie D.J., Sheppard D. (2001). Interleukin-13 induces dramatically different transcriptional programs in three human airway cell types. Am. J. Respir. Cell Mol. Biol..

[125] Zhu Z., Ma B., Zheng T., Homer R.J., Lee C.G., Charo I.F., Noble P., Elias J.A. (2002). IL-13-Induced Chemokine Responses in the Lung: Role of CCR2 in the Pathogenesis of IL-13-Induced Inflammation and Remodeling. J. Immunol..

[126] Murray L.A., Argentieri R.L., Farrell F.X., Bracht M., Sheng H., Whitaker B., Beck H., Tsui P., Cochlin K., Evanoff H.L. (2008). Hyper-responsiveness of IPF/UIP fibroblasts: Interplay between TGFβ1, IL-13 and CCL2. Int. J. Biochem. Cell Biol..

[127] Rangarajan S., Locy M.L., Luckhardt T.R., Thannickal V.J. (2016). Targeted Therapy for Idiopathic Pulmonary Fibrosis: Where to Now?. Drugs.

[128] Zhu Z., Homer R.J., Wang Z., Chen Q., Geba G.P., Wang J., Zhang Y., Elias J.A. (1999). Pulmonary expression of interleukin-13 causes inflammation, mucus hypersecretion, subepithelial fibrosis, physiologic abnormalities, and eotaxin production. J. Clin. Investig..

[129] Saito H., Papaconstantinou J., Sato H., Goldstein S. (1997). Regulation of a novel gene encoding a lysyl oxidase-related protein in cellular adhesion and senescence. J. Biol. Chem..

[130] Pascal T., Debacq-Chainiaux F., Chrétien A., Bastin C., Dabée A.F., Bertholet V., Remacle J., Toussaint O. (2005). Comparison of replicative senescence and stress-induced premature senescence combining differential display and low-density DNA arrays. FEBS Lett..

[131] Vadasz Z., Kessler O., Akiri G., Gengrinovitch S., Kagan H.M., Baruch Y., Izhak O.B., Neufeld G. (2005). Abnormal deposition of collagen around hepatocytes in Wilson’s disease is associated with hepatocyte specific expression of lysyl oxidase and lysyl oxidase like protein-2. J. Hepatol..

[132] Barry-Hamilton V., Spangler R., Marshall D., McCauley S., Rodriguez H.M., Oyasu M., Mikels A., Vaysberg M., Ghermazien H., Wai C. (2010). Allosteric inhibition of lysyl oxidase-like-2 impedes the development of a pathologic microenvironment. Nat. Med..

[133] Sgalla G., Cocconcelli E., Tonelli R., Richeldi L. (2016). Novel drug targets for idiopathic pulmonary fibrosis. Expert Rev. Respir. Med..

[134] Selman M., Ruiz V., Cabrera S., Segura L., Ramírez R., Barrios R., Pardo A. (2000). TIMP-1, -2, -3, and -4 in idiopathic pulmonary fibrosis. A prevailing nondegradative lung microenvironment?. Am. J. Physiol. Lung Cell. Mol. Physiol..

[135] Parvathaneni V., Shukla S.K., Gupta V. (2019). Emerging Therapeutic Targets and Therapies in Idiopathic Pulmonary Fibrosis. Fibrosis in Disease.

[136] Königshoff M., Kramer M., Balsara N., Wilhelm J., Amarie O.V., Jahn A., Rose F., Fink L., Seeger W., Eickelberg O. (2009). WNT1-inducible signaling protein-1 mediates pulmonary fibrosis in mice and is upregulated in humans with idiopathic pulmonary fibrosis. J. Clin. Investig..

[137] Allen J.T., Knight R.A., Bloor C.A., Spiteri M.A. (1999). Enhanced insulin-like growth factor binding protein-related protein 2 (connective tissue growth factor) expression in patients with idiopathic pulmonary fibrosis and pulmonary sarcoidosis. Am. J. Respir. Cell Mol. Biol..

[138] Wang X., Wu G., Gou L., Liu Z., Wang X., Fan X., Wu L., Liu N. (2011). A novel single-chain-Fv antibody against connective tissue growth factor attenuates bleomycin-induced pulmonary fibrosis in mice. Respirology.

[139] Grimminger F., Günther A., Vancheri C. (2015). The role of tyrosine kinases in the pathogenesis of idiopathic pulmonary fibrosis. Eur. Respir. J..

[140] Battegay E.J., Raines E.W., Seifert R.A., Bowen-Pope D.F., Ross R. (1990). TGF-β induces bimodal proliferation of connective tissue cells via complex control of an autocrine PDGF loop. Cell.

[141] Battegay E.J., Raines E.W., Colbert T., Ross R. (1995). TNF-α stimulation of fibroblast proliferation. Dependence on platelet-derived growth factor (PDGF) secretion and alteration of PDGF receptor expression. J. Immunol..

[142] Shimbori C., Bellaye P.S., Xia J., Gauldie J., Ask K., Ramos C., Becerril C., Pardo A., Selman M., Kolb M. (2016). Fibroblast growth factor-1 attenuates TGF-β1-induced lung fibrosis. J. Pathol..

[143] Shikada Y., Yonemitsu Y., Koga T., Onimaru M., Nakano T., Okano S., Sata S., Nakagawa K., Yoshino I., Maehara Y. (2005). Platelet-derived growth factor-AA is an essential and autocrine regulator of vascular endothelial growth factor expression in non-small cell lung carcinomas. Cancer Res..

[144] Ballester B., Milara J., Cortijo J. (2019). Idiopathic pulmonary fibrosis and lung cancer: Mechanisms and molecular targets. Int. J. Mol. Sci..

[145] Lee J., An Y.S., Kim M.R., Kim Y.A., Lee J.K., Hwang C.S., Chung E., Park I.C., Yi J.Y. (2016). Heat Shock Protein 90 Regulates Subcellular Localization of Smads in Mv1Lu Cells. J. Cell. Biochem..

[146] Carew R.M., Wang B., Kantharidis P. (2012). The role of EMT in renal fibrosis. Cell Tissue Res..

[147] Colunga Biancatelli R.M.L., Solopov P., Gregory B., Catravas J.D. (2020). Hsp90 inhibition and modulation of the proteome: Therapeutical implications for idiopathic pulmonary fibrosis (ipf). Int. J. Mol. Sci..

[148] Wuyts W.A., Antoniou K.M., Borensztajn K., Costabel U., Cottin V., Crestani B., Grutters J.C., Maher T.M., Poletti V., Richeldi L. (2014). Combination therapy: The future of management for idiopathic pulmonary fibrosis?. Lancet Respir. Med..

[149] Orekhov A.N., Orekhova V.A., Nikiforov N.G., Myasoedova V.A., Grechko A.V., Romanenko E.B., Zhang D., Chistiakov D.A. (2019). Monocyte differentiation and macrophage polarization. Vessel Plus.

[150] Dillingh M.R., van den Blink B., Moerland M., van Dongen M.G.J., Levi M., Kleinjan A., Wijsenbeek M.S., Lupher M.L., Harper D.M., Getsy J.A. (2013). Recombinant human serum amyloid P in healthy volunteers and patients with pulmonary fibrosis. Pulm. Pharmacol. Ther..

[151] Hynes R.O. (2002). Integrins: Bidirectional, allosteric signaling machines. Cell.

[152] Saini G., Porte J., Weinreb P.H., Violette S.M., Wallace W.A., McKeever T.M., Jenkins G. (2015). αvβ6 integrin may be a potential prognostic biomarker in interstitial lung disease. Eur. Respir. J..

[153] Cui Y., Jiang L., Yu R., Shao Y., Mei L., Tao Y. (2019). β-carboline alkaloids attenuate bleomycin induced pulmonary fibrosis in mice through inhibiting NF-kb/p65 phosphorylation and epithelial-mesenchymal transition. J. Ethnopharmacol..

[154] Jiang F., Li M., Wang H., Ding B., Zhang C., Ding Z., Yu X., Lv G. (2019). Coelonin, an Anti-Inflammation Active Component of Bletilla striata and Its Potential Mechanism. Int. J. Mol. Sci..

[155] Divya T., Velavan B., Sudhandiran G. (2018). Regulation of Transforming Growth Factor-β/Smad-mediated Epithelial-Mesenchymal Transition by Celastrol Provides Protection against Bleomycin-induced Pulmonary Fibrosis. Basic Clin. Pharmacol. Toxicol..

[156] Yang Q., Zhang P., Liu T., Zhang X., Pan X., Cen Y., Liu Y., Zhang H., Chen X. (2019). Magnesium isoglycyrrhizinate ameliorates radiation-induced pulmonary fibrosis by inhibiting fibroblast differentiation via the p38MAPK/Akt/Nox4 pathway. Biomed. Pharmacother. Biomed. Pharmacother..

[157] Li R.S., Xu G.H., Cao J., Liu B., Xie H.F., Ishii Y., Zhang C.F. (2019). α-Mangostin Ameliorates Bleomycin-Induced Pulmonary Fibrosis in Mice Partly Through Activating Adenosine 5’-Monophosphate-Activated Protein Kinase. Front. Pharmacol..

[158] Qu Y., Zhang G., Ji Y., Zhua H., Lv C., Jiang W. (2016). Protective role of gambogic acid in experimental pulmonary fibrosis in vitro and in vivo. Phytomed. Int. J. Phytother. Phytopharm..

[159] Liu Q., Chu H., Ma Y., Wu T., Qian F., Ren X., Tu W., Zhou X., Jin L., Wu W. (2016). Salvianolic Acid B Attenuates Experimental Pulmonary Fibrosis through Inhibition of the TGF-β Signaling Pathway. Sci. Rep..

[160] Chun-Bin S., Yi Y., Qin-Yi W., Yang L., Jing-Ze Y., Hai-Jing X., Si-Qi Z., Jiong H., Jing W., Fei-Yu L. (2020). The main active components of Curcuma zedoaria reduces collagen deposition in human lung fibroblast via autophagy. Mol. Immunol..

[161] Tang J., Li Y., Wang J., Wu Q., Yan H. (2019). Polydatin suppresses the development of lung inflammation and fibrosis by inhibiting activation of the NACHT domain-, leucine-rich repeat-, and pyd-containing protein 3 inflammasome and the nuclear factor-kappaB pathway after Mycoplasma pneumoniae infection. J. Cell. Biochem..

[162] Chen C., Wang Y.Y., Wang Y.X., Cheng M.Q., Yin J.B., Zhang X., Hong Z.P. (2018). Gentiopicroside ameliorates bleomycin-induced pulmonary fibrosis in mice via inhibiting inflammatory and fibrotic process. Biochem. Biophys. Res. Commun..

[163] Li C., Lu Y., Du S., Li S., Zhang Y., Liu F., Chen Y., Weng D., Chen J. (2017). Dioscin Exerts Protective Effects Against Crystalline Silica-induced Pulmonary Fibrosis in Mice. Theranostics.

[164] Park Y.J., Bang I.J., Jeong M.H., Kim H.R., Lee D.E., Kwak J.H., Chung K.H. (2019). Effects of β-Sitosterol from Corn Silk on TGF-β1-Induced Epithelial-Mesenchymal Transition in Lung Alveolar Epithelial Cells. J. Agric. Food Chem..

[165] Dong S.H., Liu Y.W., Wei F., Tan H.Z., Han Z.D. (2017). Asiatic acid ameliorates pulmonary fibrosis induced by bleomycin (BLM) via suppressing pro-fibrotic and inflammatory signaling pathways. Biomed. Pharmacother. Biomed. Pharmacother..

[166] Karkale S., Khurana A., Saifi M.A., Godugu C., Talla V. (2018). Andrographolide ameliorates silica induced pulmonary fibrosis. Int. Immunopharmacol..

[167] Mansouri M.T., Rajabi Vardanjani H., Hemmati A.A., Reza Tabandeh M., Rezaie A., Pashmforosh M., Ahmadi Angali K. (2019). Zingerone attenuates Bleomycin-Induced Pulmonary Fibrosis in Rats. Jundishapur J. Nat. Pharm. Prod..

[168] Sun S.C., Han R., Hou S.S., Yi H.Q., Chi S.J., Zhang A.H. (2020). Juglanin alleviates bleomycin-induced lung injury by suppressing inflammation and fibrosis via targeting sting signaling. Biomed. Pharmacother. Biomed. Pharmacother..

[169] Liu M.H., Lin A.H., Ko H.K., Perng D.W., Lee T.S., Kou Y.R. (2017). Prevention of Bleomycin-Induced Pulmonary Inflammation and Fibrosis in Mice by Paeonol. Front. Physiol..

[170] Pulivendala G., Bale S., Godugu C. (2020). Honokiol: A polyphenol neolignan ameliorates pulmonary fibrosis by inhibiting TGF-β/Smad signaling, matrix proteins and IL-6/CD44/STAT3 axis both in vitro and in vivo. Toxicol. Appl. Pharmacol..

[171] Li C., Yu Y., Li W., Liu B., Jiao X., Song X., Lv C., Qin S. (2017). Phycocyanin attenuates pulmonary fibrosis via the TLR2-MyD88-NF-kappaB signaling pathway. Sci. Rep..

[172] Wang Y., Dong X., Zhao N., Su X., Wang Y., Li Y., Wen M., Li Z., Wang C., Chen J. (2020). Schisandrin B attenuates bleomycin-induced pulmonary fibrosis in mice through the wingless/integrase-1 signaling pathway. Exp. Lung Res..

[173] Hemmati A.A., Pashmforosh M., Tabandeh M.R., Rezaie A., Rajabi Vardanjani H., Pipelzadeh M.H., Sistani Karampour N. (2019). Protective Effects of Morin Against Bleomycin-Induced Pulmonary Fibrosis in Mice. Jundishapur J. Nat. Pharm. Prod..

[174] Guan C., Qiao S., Lv Q., Cao N., Wang K., Dai Y., Wei Z. (2018). Orally administered berberine ameliorates bleomycin-induced pulmonary fibrosis in mice through promoting activation of PPAR-gamma and subsequent expression of HGF in colons. Toxicol. Appl. Pharmacol..

[175] Gungor H., Ekici M., Onder Karayigit M., Turgut N.H., Kara H., Arslanbas E. (2020). Zingerone ameliorates oxidative stress and inflammation in bleomycin-induced pulmonary fibrosis: Modulation of the expression of TGF-β1 and iNOS. Naunyn-Schmiedeberg’s Arch. Pharmacol..

[176] Yang F., Cao Y., Zhang J., You T., Zhu L. (2017). Glaucocalyxin A improves survival in bleomycin-induced pulmonary fibrosis in mice. Biochem. Biophys. Res. Commun..

[177] Li X.H., Xiao T., Yang J.H., Qin Y., Gao J.J., Liu H.J., Zhou H.G. (2018). Parthenolide attenuated bleomycin-induced pulmonary fibrosis via the NF-kappaB/Snail signaling pathway. Respir. Res..

[178] Fu Y., Zhao P., Xie Z., Wang L., Chen S. (2018). Oridonin Inhibits Myofibroblast Differentiation and Bleomycin-induced Pulmonary Fibrosis by Regulating Transforming Growth Factor β (TGFβ)/Smad Pathway. Med. Sci. Monit. Int. Med. J. Exp. Clin. Res..

[179] Chen L., Zhao W. (2016). Apigenin protects against bleomycin-induced lung fibrosis in rats. Exp. Ther. Med..

[180] Jiang L., Wang J., Ju J., Dai J. (2020). Salvianolic acid B and sodium tanshinone II A sulfonate prevent pulmonary fibrosis through anti-inflammatory and anti-fibrotic process. Eur. J. Pharmacol..

[181] Prashanth Goud M., Bale S., Pulivendala G., Godugu C. (2019). Therapeutic effects of Nimbolide, an autophagy regulator, in ameliorating pulmonary fibrosis through attenuation of TGF-β1 driven epithelial-to-mesenchymal transition. Int. Immunopharmacol..

[182] Yang H., Hua C., Yang X., Fan X., Song H., Peng L., Ci X. (2020). Pterostilbene prevents LPS-induced early pulmonary fibrosis by suppressing oxidative stress, inflammation and apoptosis in vivo. Food Funct..

[183] Miao K., Pan T., Mou Y., Zhang L., Xiong W., Xu Y., Yu J., Wang Y. (2020). Scutellarein inhibits BLM-mediated pulmonary fibrosis by affecting fibroblast differentiation, proliferation, and apoptosis. Ther. Adv. Chronic Dis..

[184] Kyung S.Y., Kim D.Y., Yoon J.Y., Son E.S., Kim Y.J., Park J.W., Jeong S.H. (2018). Sulforaphane attenuates pulmonary fibrosis by inhibiting the epithelial-mesenchymal transition. BMC Pharmacol. Toxicol..

[185] Liu M., Xu H., Zhang L., Zhang C., Yang L., Ma E., Liu L., Li Y. (2018). Salvianolic acid B inhibits myofibroblast transdifferentiation in experimental pulmonary fibrosis via the up-regulation of Nrf2. Biochem. Biophys. Res. Commun..

[186] Zhou X.M., Wang G.L., Wang X.B., Liu L., Zhang Q., Yin Y., Wang Q.Y., Kang J., Hou G. (2017). GHK Peptide Inhibits Bleomycin-Induced Pulmonary Fibrosis in Mice by Suppressing TGFβ1/Smad-Mediated Epithelial-to-Mesenchymal Transition. Front. Pharmacol..

[187] Ma W.H., Li M., Ma H.F., Li W., Liu L., Yin Y., Zhou X.M., Hou G. (2020). Protective effects of GHK-Cu in bleomycin-induced pulmonary fibrosis via anti-oxidative stress and anti-inflammation pathways. Life Sci..

[188] Li X., Yu H., Liang L., Bi Z., Wang Y., Gao S., Wang M., Li H., Miao Y., Deng R. (2020). Myricetin ameliorates bleomycin-induced pulmonary fibrosis in mice by inhibiting TGF-β signaling via targeting HSP90β. Biochem. Pharmacol..

[189] Yang J.Y., Tao L.J., Liu B., You X.Y., Zhang C.F., Xie H.F., Li R.S. (2019). Wedelolactone Attenuates Pulmonary Fibrosis Partly Through Activating AMPK and Regulating Raf-MAPKs Signaling Pathway. Front. Pharmacol..

[190] Xia Y., Xia Y.F., Lv Q., Yue M.F., Qiao S.M., Yang Y., Wei Z.F., Dai Y. (2016). Madecassoside ameliorates bleomycin-induced pulmonary fibrosis in mice through promoting the generation of hepatocyte growth factor via PPAR-gamma in colon. Br. J. Pharmacol..

[191] Bai L., Li A., Gong C., Ning X., Wang Z. (2020). Protective effect of rutin against bleomycin induced lung fibrosis: Involvement of TGF-β1/α-SMA/Col I and III pathway. BioFactors.

[192] Tatler A.L., Barnes J., Habgood A., Goodwin A., McAnulty R.J., Jenkins G. (2016). Caffeine inhibits TGFβ activation in epithelial cells, interrupts fibroblast responses to TGFβ, and reduces established fibrosis in ex vivo precision-cut lung slices. Thorax.

[193] Tian S.L., Yang Y., Liu X.L., Xu Q.B. (2018). Emodin Attenuates Bleomycin-Induced Pulmonary Fibrosis via Anti-Inflammatory and Anti-Oxidative Activities in Rats. Med. Sci. Monit. Int. Med. J. Exp. Clin. Res..

[194] Tao L., Cao J., Wei W., Xie H., Zhang M., Zhang C. (2017). Protective role of rhapontin in experimental pulmonary fibrosis in vitro and in vivo. Int. Immunopharmacol..

[195] Jin M., Wu Y., Wang L., Zang B., Tan L. (2016). Hydroxysafflor Yellow A Attenuates Bleomycin-induced Pulmonary Fibrosis in Mice. Phytother. Res. PTR.

[196] Li X., Lu C., Liu S., Shuaishuai L., Su C., Xiao T., Bi Z., Sheng P., Huang M., Liu X. (2018). Synthesis and discovery of a drug candidate for treatment of idiopathic pulmonary fibrosis through inhibition of TGF-β1 pathway. Eur. J. Med. Chem..

[197] Zhou X.M., Cao Z.D., Xiao N., Shen Q., Li J.X. (2016). Inhibitory effects of amines from Citrus reticulata on bleomycin-induced pulmonary fibrosis in rats. Int. J. Mol. Med..

[198] Wang L., Liu H., He Q., Gan C., Li Y., Zhang Q., Yao Y., He F., Ye T., Yin W. (2020). Galangin ameliorated pulmonary fibrosis in vivo and in vitro by regulating epithelial-mesenchymal transition. Bioorganic Med. Chem..

[199] Mehrzadi S., Hosseini P., Mehrabani M., Siahpoosh A., Goudarzi M., Khalili H., Malayeri A. (2020). Attenuation of Bleomycin-Induced Pulmonary Fibrosis in Wistar Rats by Combination Treatment of Two Natural Phenolic Compounds: Quercetin and Gallic Acid. Nutr. Cancer.

[200] Xin X., Yao D., Zhang K., Han S., Liu D., Wang H., Liu X., Li G., Huang J., Wang J. (2019). Protective effects of Rosavin on bleomycin-induced pulmonary fibrosis via suppressing fibrotic and inflammatory signaling pathways in mice. Biomed. Pharmacother. Biomed. Pharmacother..

[201] Rodrigues da Silva M., Schapochnik A., Peres Leal M., Esteves J., Bichels Hebeda C., Sandri S., Pavani C., Ratto Tempestini Horliana A.C., Farsky S.H.P., Lino-Dos-Santos-Franco A. (2018). Beneficial effects of ascorbic acid to treat lung fibrosis induced by paraquat. PLoS ONE.

[202] Oka V.O., Okon U.E., Osim E.E. (2019). Pulmonary Responses Following Quercetin Administration in Rats After Intratracheal Instillation of Amiodarone. Niger. J. Physiol. Sci. Off. Publ. Physiol. Soc. Niger..

[203] Cui Y., Xin H., Tao Y., Mei L., Wang Z. (2021). Arenaria kansuensis attenuates pulmonary fibrosis in mice via the activation of Nrf2 pathway and the inhibition of NF-kB/TGF-β1/Smad2/3 pathway. Phytother. Res. PTR.

[204] Feng F., Li N., Cheng P., Zhang H., Wang H., Wang Y., Wang W. (2020). Tanshinone IIA attenuates silica-induced pulmonary fibrosis via inhibition of TGF-β1-Smad signaling pathway. Biomed. Pharmacother. Biomed. Pharmacother..

[205] Peng L.Y., An L., Sun N.Y., Ma Y., Zhang X.W., Liu W.H., Liu B.L., Li P., Chen J. (2019). Salvia miltiorrhiza Restrains Reactive Oxygen Species-Associated Pulmonary Fibrosis via Targeting Nrf2-Nox4 Redox Balance. Am. J. Chin. Med..

[206] Bang I.J., Kim H.R., Jeon Y., Jeong M.H., Park Y.J., Kwak J.H., Chung K.H. (2019). β-Peltoboykinolic Acid from Astilbe rubra Attenuates TGF-β1-Induced Epithelial-to-Mesenchymal Transitions in Lung Alveolar Epithelial Cells. Molecules.

[207] Bahri S., Abdennabi R., Mlika M., Neji G., Jameleddine S., Ali R.B. (2019). Effect of *Phoenix dactylifera* L. Sap Against Bleomycin-Induced Pulmonary Fibrosis and Oxidative Stress in Rats: Phytochemical and Therapeutic Assessment. Nutr. Cancer.

[208] Abidi A., Robbe A., Kourda N., Ben Khamsa S., Legrand A. (2017). Nigella sativa, a traditional Tunisian herbal medicine, attenuates bleomycin-induced pulmonary fibrosis in a rat model. Biomed. Pharmacother. Biomed. Pharmacother..

[209] Chakraborty K., Dey A., Bhattacharyya A., Dasgupta S.C. (2019). Anti-fibrotic effect of black tea (*Camellia sinensis*) extract in experimental pulmonary fibrosis. Tissue Cell.

[210] Moustafa G.G., Hussein M.M.A. (2016). New insight on using aged garlic extract against toxic impacts of titanium dioxide bulk salt triggers inflammatory and fibrotic cascades in male rats. Biomed. Pharmacother. Biomed. Pharmacother..

[211] Feng F., Wang Z., Li R., Wu Q., Gu C., Xu Y., Peng W., Han D., Zhou X., Wu J. (2019). Citrus alkaline extracts prevent fibroblast senescence to ameliorate pulmonary fibrosis via activation of COX-2. Biomed. Pharmacother. Biomed. Pharmacother..

[212] Liu Q., Jiang J.X., Liu Y.N., Ge L.T., Guan Y., Zhao W., Jia Y.L., Dong X.W., Sun Y., Xie Q.M. (2017). Grape seed extract ameliorates bleomycin-induced mouse pulmonary fibrosis. Toxicol. Lett..

[213] Samareh Fekri M., Mandegary A., Sharififar F., Poursalehi H.R., Nematollahi M.H., Izadi A., Mehdipour M., Asadi A., Samareh Fekri M. (2018). Protective effect of standardized extract of *Myrtus communis* L. (myrtle) on experimentally bleomycin-induced pulmonary fibrosis: Biochemical and histopathological study. Drug Chem. Toxicol..

[214] Javad-Mousavi S.A., Hemmati A.A., Mehrzadi S., Hosseinzadeh A., Houshmand G., Rashidi Nooshabadi M.R., Mehrabani M., Goudarzi M. (2016). Protective effect of Berberis vulgaris fruit extract against Paraquat-induced pulmonary fibrosis in rats. Biomed. Pharmacother. Biomed. Pharmacother..

[215] Abidi A., Aissani N., Sebai H., Serairi R., Kourda N., Ben Khamsa S. (2017). Protective Effect of Pistacia lentiscus Oil Against Bleomycin-Induced Lung Fibrosis and Oxidative Stress in Rat. Nutr. Cancer.

[216] Qin H., Wen H.T., Gu K.J., Hu X.D., Yang T., Yan X.F., Ye T.J., Huo J.L., Hu J. (2019). Total extract of Xin Jia Xuan Bai Cheng Qi decoction inhibits pulmonary fibrosis via the TGF-β/Smad signaling pathways in vivo and in vitro. Drug Des. Dev. Ther..

[217] Liu B., Lu W., Ge H., Tang H., Li R., Zhang C. (2019). Protective Effect of the Traditional Chinese Patent Medicine Qing-Xuan Granule against Bleomycin-Induced Pulmonary Fibrosis in Mice. Chem. Biodivers..

[218] Li H., Wang Z., Zhang J., Wang Y., Yu C., Zhang J., Song X., Lv C. (2018). Feifukang ameliorates pulmonary fibrosis by inhibiting JAK-STAT signaling pathway. BMC Complementary Altern. Med..

[219] Shi W., Feng B., Xu S., Shen X., Zhang T. (2017). Inhibitory effect of compound Chuanxiong Kangxian granules on bleomycin-induced pulmonary fibrosis in rats. Biomed. Pharmacother. Biomed. Pharmacother..

[220] Chen H., Chen Q., Jiang C.M., Shi G.Y., Sui B.W., Zhang W., Yang L.Z., Li Z.Y., Liu L., Su Y.M. (2018). Triptolide suppresses paraquat induced idiopathic pulmonary fibrosis by inhibiting TGFB1-dependent epithelial mesenchymal transition. Toxicol. Lett..

[221] Chen F., Wang P.L., Fan X.S., Yu J.H., Zhu Y., Zhu Z.H. (2016). Effect of Renshen Pingfei Decoction, a traditional Chinese prescription, on IPF induced by Bleomycin in rats and regulation of TGF-β1/Smad3. J. Ethnopharmacol..

[222] Gao Y., Yao L.F., Zhao Y., Wei L.M., Guo P., Yu M., Cao B., Li T., Chen H., Zou Z.M. (2016). The Chinese Herbal Medicine Formula mKG Suppresses Pulmonary Fibrosis of Mice Induced by Bleomycin. Int. J. Mol. Sci..

[223] Liu M.W., Liu R., Wu H.Y., Li Y.Y., Su M.X., Dong M.N., Zhang W., Qian C.Y. (2016). Radix puerariae extracts ameliorate paraquat-induced pulmonary fibrosis by attenuating follistatin-like 1 and nuclear factor erythroid 2p45-related factor-2 signalling pathways through downregulation of miRNA-21 expression. BMC Complementary Altern. Med..

[224] Qian W., Cai X., Qian Q., Zhang W., Wang D. (2018). Astragaloside IV modulates TGF-β1-dependent epithelial-mesenchymal transition in bleomycin-induced pulmonary fibrosis. J. Cell. Mol. Med..

[225] Zhou Y., Liao S., Zhang Z., Wang B., Wan L. (2016). Astragalus injection attenuates bleomycin-induced pulmonary fibrosis via down-regulating Jagged1/Notch1 in lungs. J. Pharm. Pharmacol..

[226] Du J., Liang Z., Xu J., Zhao Y., Li X., Zhang Y., Zhao D., Chen R., Liu Y., Joshi T. (2019). Plant-derived phosphocholine facilitates cellular uptake of anti-pulmonary fibrotic HJT-sRNA-m7. Sci. China Life Sci..

[227] Gan W., Huang Q., Xiao G., Luo Y., Wang J., Zhang C., Liang Y., Huang N., Liao T. (2020). Modified Maimendong decoction in the treatment of patients with idiopathic pulmonary fibrosis: Study protocol for a randomized controlled trial. Medicine.

[228] Huang C., Wu X., Wang S., Wang W., Guo F., Chen Y., Pan B., Zhang M., Fan X. (2018). Combination of Salvia miltiorrhiza and ligustrazine attenuates bleomycin-induced pulmonary fibrosis in rats via modulating TNF-α and TGF-β. Chin. Med..

[229] Meng L., Zhang X., Wang H., Dong H., Gu X., Yu X., Liu Y. (2019). Yangyin Yiqi Mixture Ameliorates Bleomycin-Induced Pulmonary Fibrosis in Rats through Inhibiting TGF-β1/Smad Pathway and Epithelial to Mesenchymal Transition. Evid.-Based Complementary Altern. Med. Ecam.

[230] Bai Y., Li J., Zhao P., Li Y., Li M., Feng S., Qin Y., Tian Y., Zhou T. (2018). A Chinese Herbal Formula Ameliorates Pulmonary Fibrosis by Inhibiting Oxidative Stress via Upregulating Nrf2. Front. Pharmacol..

[231] Zhou S., Zhou Y., Yu J., Du Y., Tan Y., Ke Y., Wang J., Han B., Ge F. (2020). Ophiocordyceps lanpingensis polysaccharides attenuate pulmonary fibrosis in mice. Biomed. Pharmacother. Biomed. Pharmacother..

[232] Chen J., Shi Y., He L., Hao H., Wang B., Zheng Y., Hu C. (2016). Protective roles of polysaccharides from Ganoderma lucidum on bleomycin-induced pulmonary fibrosis in rats. Int. J. Biol. Macromol..

[233] Kim K.H., Lee S., Lee H., Shin D., Min D., Kim M., Ryu B., Kim H.W., Bae H. (2018). A standardized herbal extract PM014 ameliorates pulmonary fibrosis by suppressing the TGF-β1 pathway. Sci. Rep..

[234] Yu J.Z., Ying Y., Liu Y., Sun C.B., Dai C., Zhao S., Tian S.Z., Peng J., Han N.P., Yuan J.L. (2019). Antifibrotic action of Yifei Sanjie formula enhanced autophagy via PI3K-AKT-mTOR signaling pathway in mouse model of pulmonary fibrosis. Biomed. Pharmacother. Biomed. Pharmacother..

[235] Kim J.Y., Shin D., Lee G., Kim J.M., Kim D., An Y.M., Yoo B.R., Chang H., Kim M., Cho J. (2017). Standardized Herbal Formula PM014 Inhibits Radiation-Induced Pulmonary Inflammation in Mice. Sci. Rep..

[236] Hyo-Seok S., Hyun J.L., Choong J.L. (2016). Pyunkang-hwan (Pyunkang-Tang) ameliorates air pollutant-induced inflammatory hypersecretion of airway mucus and bleomycin- induced pulmonary fibrosis in rats. J. Tradit. Chin. Med. Chung I Tsa Chih Ying Wen Pan.

[237] Yin Z.F., Wei Y.L., Wang X., Wang L.N., Li X. (2020). Buyang Huanwu Tang inhibits cellular epithelial-to-mesenchymal transition by inhibiting TGF-β1 activation of PI3K/Akt signaling pathway in pulmonary fibrosis model in vitro. BMC Complementary Med. Ther..

[238] Zhang K., Si X.P., Huang J., Han J., Liang X., Xu X.B., Wang Y.T., Li G.Y., Wang H.Y., Wang J.H. (2016). Preventive Effects of *Rhodiola rosea* L. on Bleomycin-Induced Pulmonary Fibrosis in Rats. Int. J. Mol. Sci..

[239] Coco J.C., Ataide J.A., Sake J.A., Tambourgi E.B., Ehrhardt C., Mazzola P.G. (2021). In vitro antioxidant and wound healing properties of baru nut extract (*Dipteryx alata* Vog.) in pulmonary epithelial cells for therapeutic application in chronic pulmonary obstructive disease (COPD). Nat. Prod. Res..

[240] Su W., Liang Y., Meng Z., Chen X., Lu M., Han X., Deng X., Zhang Q., Zhu H., Fu T. (2020). Inhalation of Tetrandrine-hydroxypropyl-β-cyclodextrin Inclusion Complexes for Pulmonary Fibrosis Treatment. Mol. Pharm..

[241] Wang S.J., Han J.Y., Jia Y.M., Hu Z.Y. (2015). [High Expression of co-stimulatory molecule CD40 in silicosis patients and intervention effect of yiqi huoxue decoction]. Zhongguo Zhong Xi Yi Jie He Za Zhi Zhongguo Zhongxiyi Jiehe Zazhi Chin. J. Integr. Tradit. West. Med..

[242] Yu Y., Sun Z., Shi L., Zhang Y., Zhou Z., Zhang S., Chao E. (2016). Effects of Feiwei granules in the treatment of idiopathic pulmonary fibrosis: A randomized and placebo-controlled trial. J. Tradit. Chin. Med. Chung I Tsa Chih Ying Wen Pan.

